# Performance of a novel spectroscopy-based tool for adjuvant therapy decision-making in hormone receptor-positive breast cancer: a validation study

**DOI:** 10.1007/s10549-023-07229-y

**Published:** 2024-01-20

**Authors:** R Charles Coombes, Christina Angelou, Zamzam Al-Khalili, William Hart, Darius Francescatti, Nicholas Wright, Ian Ellis, Andrew Green, Emad Rakha, Sami Shousha, Hemmel Amrania, Chris C. Phillips, Carlo Palmieri

**Affiliations:** 1 Imperial College London, South Kensington Campus, London, SW7 2AZ UK; 2 Rush Medical College, Chicago, USA; 3 Barts Cancer Institute, London, UK; 4 Nottingham University Hospital, Nottingham, UK; 5 University of Liverpool, Liverpool, UK

**Keywords:** Breast cancer, Prognosis, Mortality, Distant recurrence

## Abstract

**Purpose:**

Digistain Index (DI), measured using an inexpensive mid-infrared spectrometer, reflects the level of aneuploidy in unstained tissue sections and correlates with tumor grade. We investigated whether incorporating DI with other clinicopathological variables could predict outcomes in patients with early breast cancer.

**Methods:**

DI was calculated in 801 patients with hormone receptor-positive, HER2-negative primary breast cancer and ≤ 3 positive lymph nodes. All patients were treated with systemic endocrine therapy and no chemotherapy. Multivariable proportional hazards modeling was used to incorporate DI with clinicopathological variables to generate the Digistain Prognostic Score (DPS). DPS was assessed for prediction of 5- and 10-year outcomes (recurrence, recurrence-free survival [RFS] and overall survival [OS]) using receiver operating characteristics and Cox proportional hazards regression models. Kaplan–Meier analysis evaluated the ability of DPS to stratify risk.

**Results:**

DPS was consistently highly accurate and had negative predictive values for all three outcomes, ranging from 0.96 to 0.99 at 5 years and 0.84 to 0.95 at 10 years. DPS demonstrated statistically significant prognostic ability with significant hazard ratios (95% CI) for low- versus high-risk classification for RFS, recurrence and OS (1.80 [CI 1.31–2.48], 1.83 [1.32–2.52] and 1.77 [1.28–2.43], respectively; all *P* < 0.001).

**Conclusion:**

DPS showed high accuracy and predictive performance, was able to stratify patients into low or high-risk, and considering its cost and rapidity, has the potential to offer clinical utility.

**Supplementary Information:**

The online version contains supplementary material available at 10.1007/s10549-023-07229-y.

## Introduction

Several risk scoring methods are available to support decision-making for adjuvant therapy in breast cancer to assess which patients could be spared or would benefit from adjuvant cytotoxic therapy, to avoid over- and under-treatment, respectively. Risk scoring tools in hormone receptor (HR)-positive and human epidermal growth factor receptor 2 (HER2)-negative early breast cancer are either based on a combination of clinicopathological factors and immunohistochemically detected tumor markers (e.g., the Nottingham Prognostic Index [NPI] and PREDICT) [1] or involve multigene expression profiles to complement pathological assessment and provide risk classification (e.g., Oncotype DX® and MammaPrint®) [2, 3]. Testing for risk prediction versus no testing is cost-effective and imparts both clinical and economic benefits [4, 5].

Genomic tests show relatively low between-test concordance due to the fact that they have been designed to evaluate the expression of different gene sets and have been clinically validated in different settings [6, 7]. Importantly, the use of genomic tests in the management of breast cancer remains relatively moderate, even in well-resourced healthcare systems [8]. Key questions remain regarding their predictive value; for example, some genomic tests do not provide prognostic information for certain subgroups of patients e.g., premenopausal women [3]. Pricing and reimbursement issues, as well as turnaround time are barriers to the wider use of genomic testing, both in the community setting and in countries with underfunded healthcare systems [9–11]. Timely assessment is particularly important given the significant inverse association between the initiation of adjuvant chemotherapy and survival in breast cancer [12, 13]. From a patient perspective, waiting for test results and for decisions regarding treatment only adds to patients’ anxiety and stress, critically underscoring the need for more rapid testing [11].

As a potential alternative or addition to genomic assays, the use of mid-infrared spectroscopy has been explored as a way to measure the concentration of specific chemical moieties present in unstained formalin-fixed tumor biopsy samples. Indeed, recent reports indicate that mid-infrared spectroscopy has significant merit in the detection of cancer with high diagnostic sensitivity and specificity [14–16].

Malignant tumors are associated with an abnormal karyotype with multiple structural and numerical aberrations of chromosomes, which can lead to aberrant mitosis and errors in chromosomal segregation. This numerical chromosomal instability or ‘aneuploidy’ has been shown to be a marker of aggressive behavior, drug resistance, and a negative prognostic factor in several tumor types, including breast cancer [17–21]. However, sample preparation challenges, sample quality control and expensive equipment have hindered the adoption of ploidy measurements using flow cytometry in the routine clinical setting [22].

**Fig. 1:**
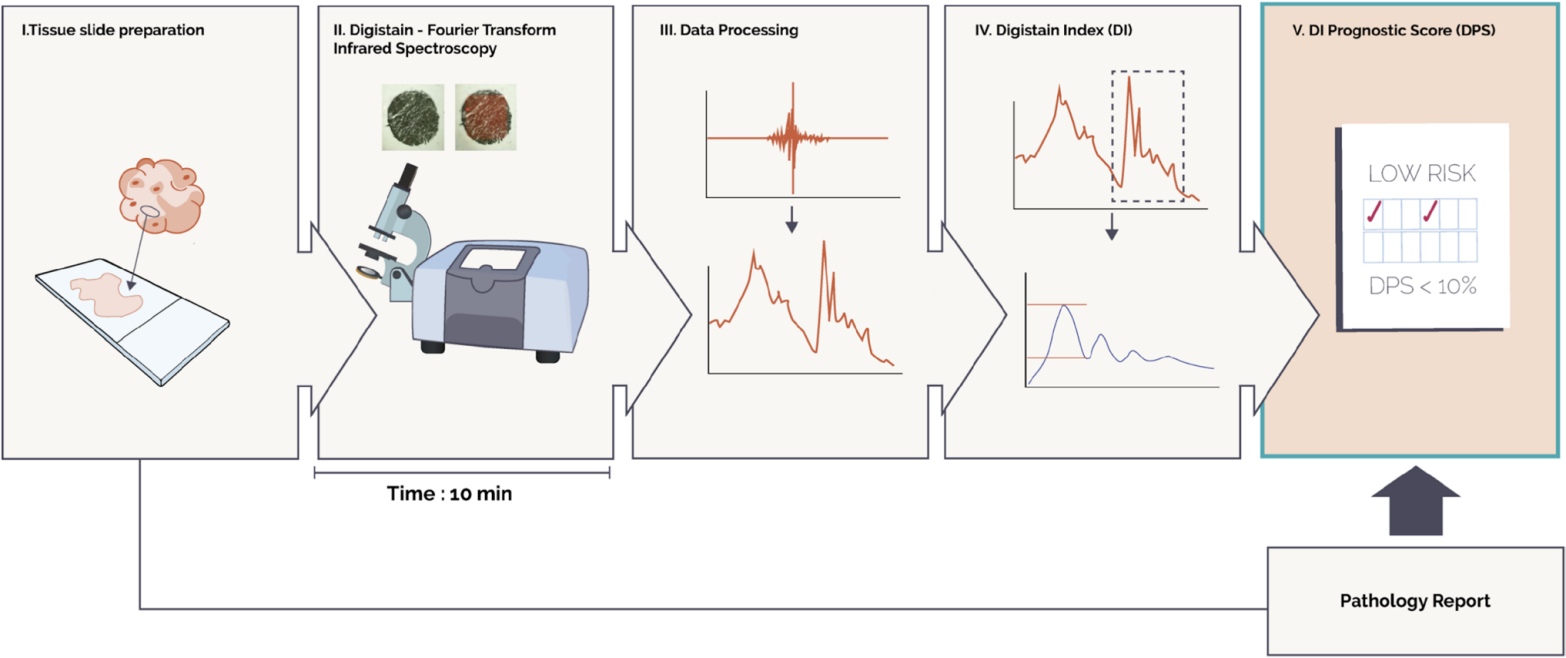
Flow diagram of the Digistain protocol. Digistain procedure: *(1)* Tissue slides are prepared from formalin-fixed paraffin embedded tumor biopsies following standard processes as for H&E staining and sections are mounted onto an infrared-transmitting calcium fluoride microscope slide. *(2)* After deparaffinization, slides are placed under the microscope for optical imaging. Light signals are converted into electric signals through a mercury cadmium telluride detector generating an interferogram. *(3)* The interferogram is then converted into an absorption spectrum, a plot of absorbance versus wavenumber, through Fourier Transformation performed by OPUS software. *(4)* Analysis of the peaks and baselines of absorbance defines the Digistain Index (DI), which is quantified using MATLAB code. *(5) *The Digistain Prognostic Score (DPS) is derived from the DI and data extracted from a standard histopathology report

The ‘Digistain Index’ (DI) has been developed that reflects the level of aneuploidy within a tumor [23, 24]. The method uses mid-infrared spectroscopy to measure the concentrations of chemical moieties, such as phosphate and amide, to determine the nuclear-to-cytoplasmic chemical ratio in the cellular content of malignant tissue. Using proprietary software, DI provides a rapid, reproducible, quantitative score of aneuploidy-related changes as an objectively obtained physical measurement from cells in unstained formalin-fixed tumor biopsy sections at the same time as routine hematoxylin and eosin (H&E) staining, without tissue maceration or other special handling (Fig. 1) [24, 25]. We have previously demonstrated that the DI univariately correlates with tumor grade (*P* = 0.0007) and shows promise for risk stratification when a preliminary defined cut-off is applied (*P* = 0.02 log-rank test) in patients with breast cancer [25].

Here we describe the validation of Digistain Prognostic Score (DPS), developed by incorporating DI with clinicopathological features, to predict 5- and 10-year recurrence-free survival (RFS), recurrence and overall survival (OS) in a well-characterized cohort of patients with early HR-positive HER2-negative breast cancer who had received systemic adjuvant endocrine therapy, but no chemotherapy.

## Methods

### Patients

In this retrospective study, 801 randomly selected non-consecutive patients aged ≤ 70 years, with HR-positive HER2-negative primary operable breast cancer and ≤ 3 positive lymph nodes (LN) were identified through multidisciplinary team records at Nottingham City Hospital (UK) where they were treated between 1998 and 2006. All tumors were less than 5 cm diameter on clinical/pre-operative measurement. All patients were treated with systemic endocrine therapy (tamoxifen). Patients with NPI > 3.4 also received goserelin for ovarian function suppression if premenopausal. No patients received adjuvant or neoadjuvant chemotherapy. All patients received some form of adjuvant radiotherapy post-surgery and in place of axillary clearance for node-positive patients. Patients were excluded in the case of no tumor or ductal carcinoma in situ, if chemotherapy or neoadjuvant therapy was received, if estrogen receptor (ER) status was unknown or negative, and if no long-term follow-up was available.

### Sample preparation and Digistain procedure

Tissues samples were processed and analyzed as described previously [23, 25] and in the Supplementary Information. Briefly, samples were de-identified and two adjacent sections, each ~ 4 μm thick, were cut from each tissue microarray block. One section was H&E stained and graded according to the Elston–Ellis method for NPI scoring. The second serial section was mounted on an infrared-transmitting calcium fluoride microscope slide and, after deparaffinization, was placed in a Bruker Vertex70 Infrared Spectrometer equipped with a Hyperion 2000 microscope. The microscope aperture was set to sample an area of 500 μm by 500 μm (smaller than the area of the core). The aperture was centered over each core and an average of 64 interferograms was then recorded for each unstained core section on the slide. The resulting averaged interferogram for each sample was Fourier Transformed and thus converted to an absorption spectrum using Bruker’s OPUS software. DI was quantified using proprietary software written in MATLAB version R2022b (Fig. 1).

Considering that DNA aneuploidy has been shown to correlate with a high malignancy grade, frequent mitoses and a high degree of nuclear pleomorphism, as well as the difficulties in assessing aneuploidy in tissue sections, we used pleomorphism as a surrogate for aneuploidy and examined its relationship to DI.

### Statistical analysis

An analysis of variance model (type III tests of fixed effects) was used to assess the relationship between DI and pleomorphism, where the degree of nuclear pleomorphism was reported as a subcomponent score in the histological grading of the tumor samples.

Following best practices [26], the DPS was generated for each tissue sample using a multivariable proportional hazards model constructed from the following covariates: patient age at diagnosis, LN status, tumor grade and tumor size, and DI (see Supplementary Information). To define the continuous relationship between DPS and each clinical outcome (risk of recurrence, probability of RFS and OS), the data were fitted by a time-varying, piecewise, log-hazard ratio model with all covariables. All clinical outcomes were defined according to the STEEP criteria for adjuvant trials where recurrence is defined to include distant, local, and ipsilateral recurrence. For each clinical outcome, data were analyzed from the date of surgery to the time of the first event or date on which data were censored. To eliminate bias, patient follow-up data were released by the Nottingham Tissue Bank after the Digistain procedure and were analyzed by an independent statistician.

Classification of patients into two groups (high versus low-risk) was made using DPS cut-offs that were chosen prospectively to correspond with a 10% rate of recurrence, disease-specific death or death. A multivariable logistic model modelling the event (as defined by the STEEP Criteria: recurrence, disease-specific death or death, and death) after 5 or 10 years was generated. This was done using the Cox model and examining the predicted risk of an event against outcomes at defined time horizons of 5 and 10 years. Receiver operator characteristics (ROC) curves were constructed and area under the ROC curve (AUC) calculated, with an AUC of 1 representing perfect prediction and 0.5 representing random prediction (i.e., a test of no value). Given the cohort was of relatively low inherent risk, there were a limited number of events and therefore K-fold cross validation [27, 28] was used to validate the performance metrics of the high/low-risk classification. This allowed the model to train on multiple training sets and avoid overfitting, where the validation set originated from the same institution as the training set.

To examine the ability of DPS to predict clinical outcome, separate analyses tested the hypotheses that the proportion of patients with better clinical outcomes i.e., (a) free of recurrence, (b) not recurred and alive, or (c) are still alive at 10 years would be higher in the low-risk group than in the high-risk group. The Kaplan–Meier log-rank test was used to evaluate if the difference between the two risk classifications was statistically significant.

Statistical analyses were conducted for the total patient group and four subgroups: patients with 0 or 1–3 positive LN, and pre- and post-menopausal patients since these factors have an impact on adjuvant treatment decisions. Age below 50 years and above 60 years was used as a surrogate for determining menopausal status to mitigate inclusion of perimenopausal patients and data from patients between these ages were excluded from the subgroup analyses due to indeterminate menopausal status. The model parameters were kept consistent in evaluating the performance for each subgroup.

A *P* value of less than 0.05 (two-sided) was considered to be a significant result. All statistical analyses were performed with Python version 3.9.

## Results

### Study cohort, clinicopathological data and outcomes

Of the 801 patients, 548 (68.41%) were LN-negative, 244 (30.46%) were premenopausal and 296 (36.95%) were postmenopausal by age (Table 1). For the total population, median age at diagnosis was 53 years and median tumor size was 1.6 cm. Most patients had a ductal tumor (85.02%), while much smaller proportions had lobular tumors (10.36%) or special-type cancers (4.37%). At the time of diagnosis, 46.32% had a good NPI score (> 2.4 and ≤ 3.4), 46.32% had a moderate NPI score (> 3.4 and ≤ 5.4), and 7.24% had a poor NPI score (> 5.4) (with data missing for one patient). The median length of follow-up from diagnosis to last follow-up was 12.7 years (range, 0.9 to 19 years), with 90% of patients experiencing no recurrence in the 10 years from diagnosis.

**Table 1:** Summary of patient and tumor characteristics in the total population and by subgroup

	Total population	Lymph node negative	Lymph node positive	Age ≤ 50 years (premenopausal)	Age ≥ 60 years (postmenopausal)
Median age (IQR), years	56 (49–63)	57 (50–64)	53 (47–61)	45 (41–48)	65 (62–68)
Median tumor size (IQR), cm	1.6 (1.2–2.2)	1.4 (1.1–1.9)	2.1 (1.5–2.7)	1.8 (1.4–2.5)	1.5 (1.1–2.0)
Tumor type, *n* (%)					
Ductal (including mixed)	681 (85.02)	464 (84.67)	217 (85.77)	215 (87.40)	247 (82.61)
Lobular	83 (10.36)	54 (9.85)	29 (11.46)	22 (8.94)	38 (12.71)
Mixed no special type and lobular	1 (0.12)	1 (0.18)	0 (0.00)	0 (0.00)	1 (0.33)
Special type	35 (4.37)	29 (5.29)	6 (2.37)	8 (3.25)	13 (4.35)
Tubular	1 (0.12)	0 (0.00)	1 (0.40)	1 (0.41)	0 (0.00)
Tumor grade, *n* (%)					
1	168 (20.97)	134 (24.45)	34 (13.44)	34 (13.82)	75 (25.08)
2	395 (49.31)	267 (48.72)	128 (50.59)	106 (43.09)	148 (49.50)
3	238 (29.71)	147 (26.82)	91 (35.97)	106 (43.09)	76 (25.42)
Lymph node status, *n* (%)					
Negative	548 (68.41)	548 (100.00)	0 (0.00)	148 (60.16)	226 (75.59)
1–3 positive	253 (31.59)	0 (0.00)	253 (100.00)	98 (39.84)	73 (24.41)
Pleomorphism, *n* (%)					
1	17 (2.12)	14 (2.55)	3 (1.19)	3 (1.22)	9 (3.01)
2	300 (37.45)	219 (39.96)	81 (32.02)	63 (25.61)	131 (43.81)
3	477 (59.55)	309 (56.39)	168 (66.40)	176 (71.54)	157 (52.51)
Missing data	7 (0.87)	6 (1.09)	1 (0.40)	4 (1.63)	2 (0.67)
Nottingham prognostic index, n (%)					
Good prognostic score	371 (46.32)	346 (63.14)	25 (9.88)	79 (32.11)	159 (53.18)
Moderate prognostic score	371 (46.32)	202 (36.86)	169 (66.80)	138 (56.10)	125 (41.81)
Poor prognostic score	58 (7.24)	0 (0.00)	58 (22.92)	28 (11.38)	15 (5.02)
Missing data	1 (0.12)	0 (0.00)	1 (0.40)	1 (0.41)	0 (0.00)
Recurrence					
No	642 (80.15)	459 (83.76)	183 (72.33)	190 (77.24)	240 (80.27)
Yes	159 (19.85)	89 (16.24)	70 (27.67)	56 (22.76)	59 (19.73)

### DI distribution and pleomorphism

The mean DI value was 0.9 (standard deviation, 0.09; median, 1.0) with a minimum value of 0.58 and a maximum of 1.31. The distribution of DI values was slightly more skewed than would be expected from a variable showing normal distribution, even when log or inverse transformation was applied. Nevertheless, mean and median DI were very close and further analyses considered DI as a normally distributed variable. Most tumors were pleomorphism grade 3 (59.55%) or grade 2 (37.45%), with a small proportion of grade 1 (2.12%) (Fig. 1). DI showed a borderline statistically significant relationship with pleomorphism (*F* = 2.92, *P* = 0.053).

### Accuracy of DI-based risk prognostication

In a Cox model, there were significant associations (all *P* < 0.001) between OS and tumor grade (hazard ratio 1.81; 95% CI 1.46–2.30), tumor size (1.37; 1.19–1.57), age (1.04; 1.03–1.06), and LN stage (1.78; 1.34–2.36). However, DI exhibited the highest hazard ratio of 4.49 (95% CI 1.08–18.67), albeit with a *P* value of 0.039 (Table 2). Similar findings were noted for RFS (Table 2). Direct comparison is possible as the data sets were normalized with respect to the mean and standard deviation of each variable (Supplemental Information). It is worth noting that although grading is generally associated with high levels of interobserver error in this cohort, the Cox model indicated a statistically significant link with recurrence (data not shown).

**Table 2:** Cox proportional hazard model for estimating the contribution of variables to predict overall survival and recurrence-free survival in the total population (*N* = 801)

Variable	Overall survival		Recurrence-free survival	
	Hazard ratio (95% CI)	*P* value	Hazard ratio (95% CI)	*P* value
Grade	1.81 (1.46–2.30)	< 0.001	1.60 (1.30–1.95)	< 0.001
Size	1.37 (1.19–1.57)	< 0.001	1.27 (1.12–1.43)	< 0.001
Age	1.04 (1.03–1.06)	< 0.001	1.03 (1.01–1.04)	< 0.001
Digistain Index	4.49 (1.08–18.67)	0.039	4.41 (1.36–14.27)	0.013
Lymph node stage	1.78 (1.34–2.36)	< 0.001	1.48 (1.14–1.97)	< 0.001

When DI was combined with the other variables to generate the DPS, the AUC values for the ROC curves obtained at the 5-year analysis for all three clinical outcomes examined and across all patient groups was consistently high for DPS (Table 3). The AUC values for the ROC curves obtained at the 10-year analysis were lower than those at 5 years but remained at a high predictive level across all clinical outcomes. AUC values were highest in the total population than any of the four subgroups examined. In the total population, the AUC for RFS and recurrence were the same, 0.81 and 0.75 at 5 and 10 years, respectively, with AUCs for OS of 0.77 and 0.69 at 5 and 10 years, respectively (Table 3, Figure S1). In the four subgroups, AUC values were similar for all outcomes ranging from 0.67 to 0.80 and 0.60 to 0.75 for 5 and 10 years, respectively (Table 3, Figures S2–S5).

**Table 3:** Digistain accuracy (NPV, PPV, and AUC under ROC curve) for prediction of risk scoring for 5-year and 10-year clinical outcomes in the total population and by subgroup

	Total population		Lymph node negative		Lymph node positive		Age ≤ 50 years (premenopausal)		Age ≥ 60 years (postmenopausal)	
	5 years	10 years	5 years	10 years	5 years	10 years	5 years	10 years	5 years	10 years
AUC										
RFS	0.81	0.75	0.71	0.70	0.77	0.73	0.74	0.62	0.71	0.74
Recurrence	0.81	0.75	0.71	0.69	0.77	0.73	0.75	0.61	0.72	0.75
OS	0.77	0.69	0.67	0.62	0.75	0.73	0.80	0.60	0.70	0.66
NPV										
RFS	0.99	0.94	0.99	0.95	0.98	0.89	0.98	0.91	0.98	0.92
Recurrence	0.99	0.94	0.99	0.95	0.98	0.91	0.99	0.91	0.98	0.92
OS	0.97	0.90	0.97	0.90	0.98	0.87	0.97	0.92	0.96	0.84
PPV										
RFS	0.06	0.15	0.02	0.10	0.11	0.20	0.08	0.15	0.05	0.14
Recurrence	0.07	0.15	0.02	0.11	0.11	0.20	0.09	0.14	0.06	0.14
OS	0.08	0.18	0.03	0.12	0.14	0.24	0.07	0.16	0.08	0.21

*AUC* area under the curve, *NPV* negative predictive value, *OS* overall survival, *PPV* positive predictive value, *RFS* recurrence-free survival, *ROC* receiver operating characteristics curve

Across all groups and for all clinical outcomes, there were similar trends of low (< 0.21) positive predictive values (PPV) and high (> 0.84) negative predictive values (NPV) (Table 3). At 5 years, PPV ranged from 0.02 to 0.09. At 10 years, they were somewhat higher ranging from 0.10 to 0.24. NPV were high across all three clinical outcomes, ranging from 0.96 to 0.99 at 5 years and 0.84 to 0.95 at 10 years. Importantly, among the subgroups analyzed, risk stratification accuracy with DPS was significant with an NPV of 0.95 for the prediction of 10-year recurrence and 0.95 for 10-year RFS in the LN-negative subgroup.

### DPS and clinical outcomes

**Fig. 2:**
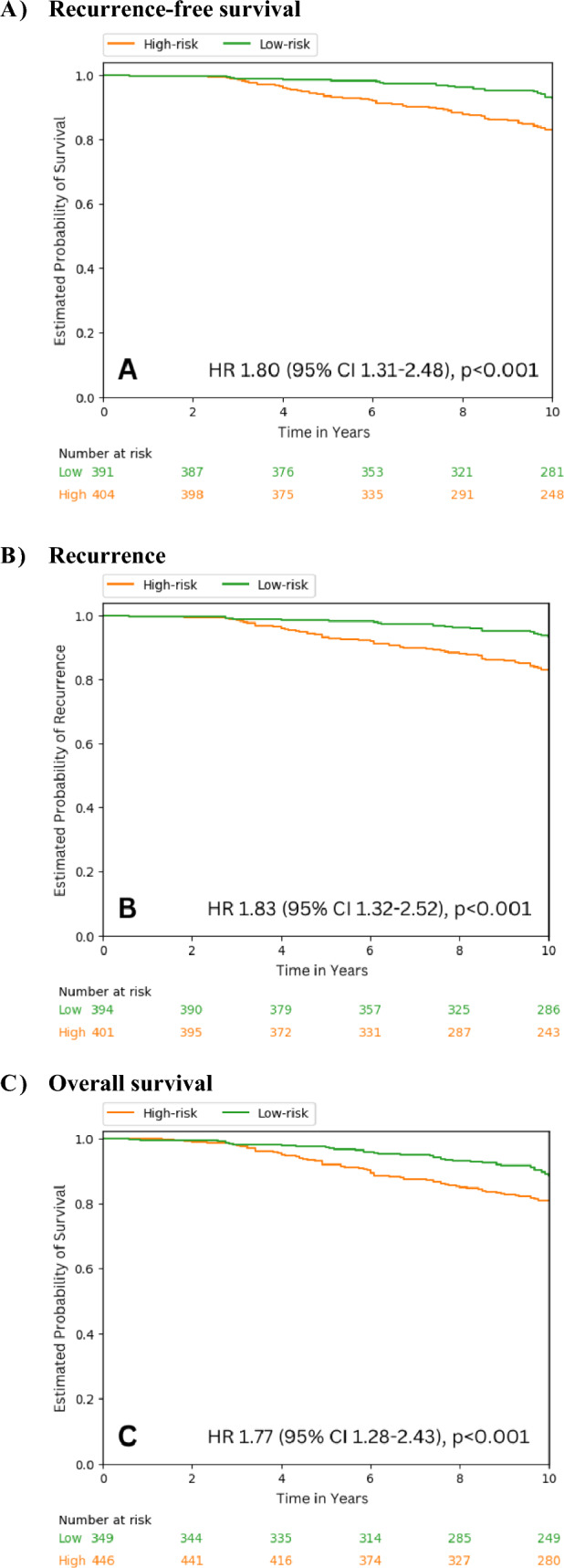
Kaplan–Meier curves indicating the event distribution over time for clinical outcomes based on Digistain Prognosis Score classification for high and low-risk in the total population

In the total population, after classifying patients into high and low-risk using a prospectively chosen cut-off point for DPS, the Kaplan–Meier estimate for the proportion of patients in the low-risk category who were free of recurrence at 10 years after diagnosis was 49.6%, while the proportion of patients in the low-risk category who had not recurred and who were still alive was 49.2% and 86.7%, respectively (Table 4, Fig. 2). For the clinical outcomes studied, approximately half of the patients in all four groups were classified as low-risk. As expected, the percentage of the low-risk patients was slightly higher in the LN-negative subgroup for recurrence and RFS (Table 4). For OS, 55.7% of patients were classified as low-risk compared with 18.07% in the LN-positive subgroup. As may be expected, the premenopausal younger patients had higher rates of recurrence compared with the postmenopausal older patients; 53.3% of patients were classified as high-risk in the younger patient subgroup compared with 42.6% in the older patient subgroup.

**Table 4:** Hazard ratio for recurrence-free survival, recurrence, and overall survival in the total population and subgroups according to Digistain Prognostic Score-based risk multivariable model high-low classification

	Total population	Lymph node negative	Lymph node positive	Age ≤ 50 years (premenopausal)	Age ≥ 60 years (postmenopausal)
	Hazard ratio (95%CI)				
Recurrence-free survival	1.80 (1.31–2.48) *P* < 0.001	1.63 (1.08–2.48) *P* = 0.021	1.48 (0.81–2.71) *P* = 0.202	1.91 (1.11–3.28) *P* = 0.019	1.99 (1.18–3.34) *P* < 0.001
N–low-risk	391 (49.18%)	335 (61.14%)	56 (22.49%)	116 (47.54%)	166 (56.08%)
N—high-risk	404 (50.82%)	211 (38.64%)	193 (77.51%)	128 (52.46%)	130 (43.91%)
Recurrence	1.83 (1.32–2.52) *P* < 0.001	1.61 (1.06–2.47) *P* = 0.027	1.55 (0.83–2.89) *P* = 0.168	2.06 (1.18–3.60) *P* = 0.011	2.22 (1.31–3.74) *P* = 0.002
N—low-risk	394 (49.56%)	341 (62.45%)	53 (21.29%)	114 (46.72%)	170 (57.43%)
N—high-risk	401 (50.44%)	205 (37.55%)	196 (78.71%)	130 (53.28%)	126 (42.56%)
Overall survival	1.77 (1.28–2.43) *P* < 0.001	1.38 (0.92–2.07) *P* = 0.124	1.67 (0.88–3.15) *P* = 0.116	2.16 (1.09–4.28) *P* = 0.028	1.66 (1.08–2.57) *P* = 0.022
N—low-risk	349 (43.90%)	304 (55.68%)	45 (18.07%)	100 (40.98%)	147 (49.66%)
N—high-risk	446 (56.10%)	242 (44.32%)	204 (81.93%)	144 (59.02%)	149 (50.34%)

In the total population, the hazard ratio for DPS was statistically significant (*P* < 0.001) for low- versus high-risk classification for all three clinical outcomes (1.80, 1.83 and 1.77 for RFS, recurrence and OS, respectively) (Table 4, Fig. 2). In the subgroups analyzed, DPS showed statistically significant risk prognostication for RFS and recurrence in the LN-negative subgroup, and for all three outcomes in the premenopausal and postmenopausal subgroups (Table 4, Figure S6). In general, stratifying the menopausal subgroups further by LN stage did not produce significant results, which may be due to the low number of events and patients (Table S1).

## Discussion

In the current study, we utilized a novel technology to determine prognosis in early-stage breast cancer using tissue microarrays from a well-characterized series. DI provides a measure of aneuploidy [23], which has remained unexplored as a prognostic marker mainly due to the limitations of flow cytometry and lack of assays that are amenable to routine clinical settings [22, 29, 30]. Aneuploidy-related changes have been shown to be independent prognostic markers in multivariable analyses of cohorts of cancer patients including breast cancer [19, 20, 31]. To support the link between aneuploidy and DI, we examined pleomorphism, which has been demonstrated to correlate to aneuploidy [32], and found that DI shows a close to statistically significant relationship to pleomorphism. Digistain-based tools may have an advantage over gene-expression analysis in that transcription does not necessarily correlate with protein synthesis, and as such, genomics-based prognostic tools may not fully assess tumor features and aggressiveness.

Our multivariable model incorporating DI together with age, tumor grade, tumor size, and LN status showed accuracy of risk prediction well in the range of clinical value. It is notable that whilst DI was significantly associated with both overall survival and RFS, the 95% CIs for the hazard ratios were wide. Digistain relies on recording an infrared absorption spectrum from a preselected region of interest and heterogeneity effects may be partly responsible for the spread in CIs, as well as the relatively low event ratio inherent to the low-risk cohort. Wide CIs have also been seen with other prognostic markers, such as MammaPrint where the hazard ratio was 4.6 and 95% CIs were 2.3–9.2 [33]. The prognostic magnitude of the composite score (DPS) appeared to reduce compared with DI alone, with a final hazard ratio of 1.8 for RFS. This is expected based on the contribution of each of the variables, which are required to produce a robust prognostic model that accounts for occasional non-performance of any individual clinically relevant predictor. Inclusion of DI into a multivariable model resulted in a prognostic model that further stratified an inherently low-risk population, and is therefore considered to be of clinical relevance and utility.

Information on the accuracy of commercial genomic tools is not readily available and cross-comparisons may also be confounded by nuances of the patient population and methodology in such studies [34]. Nevertheless, a study conducted by the TRANSBIG Consortium to validate MammaPrint in LN-negative breast cancer and where the Adjuvant! Software was used to initially assign risk groups, reported that the AUC for predicting 5-year time to distant metastasis was 0.68 and 0.65 for MammaPrint and Adjuvant! Software, respectively, and 0.64 and 0.57 for predicting 10-year OS, respectively [35]. In our study, risk prediction with DPS showed similar if not better results in a comparable subgroup population.

The performance of this DI-based risk scoring tool is highly promising in terms of NPV, with values above 97% for predicting RFS, recurrence or OS at 5 years and over 89% at 10 years. These values are similar to those reported for MammaPrint and other tools [36–38] where clinical indication is defined as the identification of low-risk patients and therefore the priority is a high NPV using a 10% risk cut-off. As such, the relatively low PPVs are expected and in line with prognostic markers validated on patient cohorts not treated with cytotoxic therapy. Given the fact that these prognostic tests are intended to mitigate overtreatment with chemotherapy, the high NPV allows for the identification of true low-risk patients who may safely de-escalate treatment.

In total, 90% of the study population experienced no recurrence in the 10 years from diagnosis. The study was performed on a cohort who received no chemotherapy and was a low-risk population by design. Included patients were treated between 1998 and 2006 and are somewhat atypical of a similar contemporary population. However, there is still a current need for an accessible method to ensure most low-risk patients are spared unnecessary treatments, given the unaffordability of genomic risk profiling tests and the fact they are generally reserved for node negative cases with NPI > 3.4 [39].

Using the DPS, hazard ratios for all three outcomes were statistically significant for low- versus high-risk classification. The ability to stratify patients with a reliable, rapid and accessible test across real-life clinical settings has the potential to help guide decision-making regarding subsequent adjuvant therapies as an alternative to existing tools or additionally in intermediate-risk patients. Unlike genomic assays, Digistain does not involve costly reagents and employs widely available and inexpensive infrared spectrometers. Easily incorporated into pathologists’ workflow, Digistain is performed on routinely processed unstained tissue sections, with no special tissue handling regimens and reduced variability due to processing and RNA yield. Of note, it is not susceptible to the subjectivity issues associated with prognostic tools based on clinicopathological features [25].

In our subgroup analysis, we found that AUC values were lower in the subgroups than in the total population, which may be due to the smaller numbers of patients available at the 10-year time point, although AUC values remained reasonably high. In the LN-negative subgroup, accuracy was high for the prediction of 10-year recurrence and RFS. Furthermore, DPS showed significant risk prognostication in the LN-negative subgroup (for RFS and recurrence), and in premenopausal patients (all three outcomes). These initial results are promising given the limited use of other biomarkers in these underserved patient groups [3] and since current UK guidance recommends the use of prognostic tests only for node negative cases [39]. Further investigations of DPS with larger samples are now warranted by menopausal status (determined not solely based on age) and in those receiving versus not receiving adjuvant therapies. Patients were included in the cohort if their tumors were reported as ER+ and there was no information on specific levels of ER reported from immunohistochemical staining. It would be useful to explore the prognostic potential of ER sensitivity e.g., ER > 9% versus 1–10% in a future study with DPS, although ER levels are most commonly reported as a binary variable, particularly in under-resourced settings. DPS performance could also be analyzed in relation to TNM staging and staging combined with NPI or tumor grade. Additional multicenter studies (to avoid inclusion bias) are in progress, and we are currently investigating the prognostic impact of the number of positive LN involved. We are also exploring the ability of DPS to predict chemotherapy benefit and conducting cost-effectiveness analyses on the value of DPS across different clinical characteristics and risk groups.

To conclude, we have demonstrated that DPS is able to classify HR-positive HER2-negative primary operable breast cancer and ≤ 3 positive LN into low or high-risk with similar accuracy and predictive performance as that reported for other risk stratification tools. With currently over-burdened healthcare systems and the need to improve global cancer care inequalities, the ability to provide low-cost, rapid, and widely accessible prognostic testing suggests that Digistain may have the potential for significant clinical utility.

### Supplementary Information

Below is the link to the electronic supplementary material.

Supplementary file1 (DOCX 1873 kb)

## Data Availability

Data supporting the findings of this study are available upon request from the corresponding author.

## Abbreviations

AUC: Area under the ROC curve
CI: Confidence interval
DI: Digistain Index
DPS: Digistain Prognostic Score
ER: Estrogen receptor
H&E: Hematoxylin and eosin
HER2: Human epidermal growth factor receptor 2
HR: Hormone receptor
LN: Lymph node
NPI: Nottingham Prognostic Index
NPV: Negative predictive value
OS: Overall survival
PPV: Positive predictive value
RFS: Recurrence-free survival
ROC: Receiver operator characteristics

## References

[1] Gunda A, Eshwaraiah MS, Gangappa K, Kaur T, Bakre MM (2022). A comparative analysis of recurrence risk predictions in ER+/HER2- early breast cancer using NHS Nottingham Prognostic Index, PREDICT, and CanAssist Breast. Breast Cancer Res Treat.

[2] Markopoulos C, Hyams DM, Gomez HL, Harries M, Nakamura S, Traina T, Katz A (2020). Multigene assays in early breast cancer: Insights from recent phase 3 studies. Eur J Surg Oncol.

[3] Andre F, Ismaila N, Allison KH, Barlow WE, Collyar DE, Damodaran S, Henry NL, Jhaveri K, Kalinsky K, Kuderer NM, Litvak A, Mayer EL, Pusztai L, Raab R, Wolff AC, Stearns V (2022). Biomarkers for adjuvant endocrine and chemotherapy in early-stage breast cancer: ASCO guideline update. J Clin Oncol.

[4] Hannouf MB, Zaric GS, Blanchette P, Brezden-Masley C, Paulden M, McCabe C, Raphael J, Brackstone M (2020). Cost-effectiveness analysis of multigene expression profiling assays to guide adjuvant therapy decisions in women with invasive early-stage breast cancer. Pharmacogenomics J.

[5] Assi HI, Alameh IA, Khoury J, Abdul Halim N, El Karak F, Farhat F, Berro J, Sbaity E, Charafeddine M, Tfayli A, Salem Z, El Saghir N (2020). Impact of commercialized genomic tests on adjuvant treatment decisions in early stage breast cancer patients. J Oncol.

[6] Hillyar C, Rizki H, Abbassi O, Miles-Dua S, Clayton G, Gandamihardja T, Smith S (2020). Correlation between Oncotype DX, PREDICT and the Nottingham Prognostic Index: implications for the management of early breast cancer. Cureus.

[7] Bartlett JM, Bayani J, Marshall A, Dunn JA, Campbell A, Cunningham C, Sobol MS, Hall PS, Poole CJ, Cameron DA, Earl HM, Rea DW, Macpherson IR, Canney P, Francis A, McCabe C, Pinder SE, Hughes-Davies L, Makris A, Stein RC, OPTIMA TMG,  (2016). Comparing breast cancer multiparameter tests in the OPTIMA Prelim trial: no test is more equal than the others. J Natl Cancer Inst.

[8] Lieu TA, Ray GT, Prausnitz SR, Habel LA, Alexeeff S, Li Y, Ramsey SD, Phelps CE, Chawla N, O'Neill C, Mandelblatt SJS (2017). Oncologist and organizational factors associated with variation in breast cancer multigene testing. Breast Cancer Res Treat.

[9] Ibraheem A, Olopade OI, Huo D (2020). Propensity score analysis of the prognostic value of genomic assays for breast cancer in diverse populations using the National Cancer Data Base. Cancer.

[10] Sechrist H, Glasgow A, Bomeisl P, Gilmore H, Harbhajanka A (2020). Concordance of breast cancer biomarker status between routine immunohistochemistry/in situ hybridization and Oncotype DX qRT-PCR with investigation of discordance, a study of 591 cases. Hum Pathol.

[11] Ersek JL, Black LJ, Thompson MA, Kim ES (2018). Implementing precision medicine programs and clinical trials in the community-based oncology practice: barriers and best practices. Am Soc Clin Oncol Educ Book.

[12] Chavez-MacGregor M, Clarke CA, Lichtensztajn DY, Giordano SH (2016). Delayed initiation of adjuvant chemotherapy among patients with breast cancer. JAMA Oncol.

[13] Yung R, Ray RM, Roth J, Johnson L, Warnick G, Anderson GL, Kroenke CH, Chlebowski RT, Simon MS, Fung C, Pan K, Wang D, Barrington WE, Reding KW (2020). The association of delay in curative intent treatment with survival among breast cancer patients: findings from the Women’s Health Initiative. Breast Cancer Res Treat.

[14] Su KY, Lee WL (2020). Fourier transform infrared spectroscopy as a cancer screening and diagnostic tool: a review and prospects. Cancers (Basel).

[15] Sala A, Anderson DJ, Brennan PM, Butler HJ, Cameron JM, Jenkinson MD, Rinaldi C, Theakstone AG, Baker MJ (2020). Biofluid diagnostics by FTIR spectroscopy: a platform technology for cancer detection. Cancer Lett.

[16] Cameron JM, Brennan PM, Antoniou G, Butler HJ, Christie L, Conn JJA, Curran T, Gray E, Hegarty MG, Jenkinson MD, Orringer D, Palmer DS, Sala A, Smith BR, Baker MJ (2022). Clinical validation of a spectroscopic liquid biopsy for earlier detection of brain cancer. Neurooncol Adv.

[17] Lukow DA, Sausville EL, Suri P, Chunduri NK, Wieland A, Leu J, Smith JC, Girish V, Kumar AA, Kendall J, Wang Z, Storchova Z, Sheltzer JM (2021). Chromosomal instability accelerates the evolution of resistance to anti-cancer therapies. Dev Cell.

[18] Xu J, Huang L, Li J (2016). DNA aneuploidy and breast cancer: a meta-analysis of 141,163 cases. Oncotarget.

[19] Pinto AE, Pereira T, Santos M, Branco M, Dias A, Silva GL, Ferreira MC, André S (2013). DNA ploidy is an independent predictor of survival in breast invasive ductal carcinoma: a long-term multivariate analysis of 393 patients. Ann Surg Oncol.

[20] Hieronymus H, Murali R, Tin A, Yadav K, Abida W, Moller H, Berney D, Scher H, Carver B, Scardino P, Schultz N, Taylor B, Vickers A, Cuzick J, Sawyers CL (2018). Tumor copy number alteration burden is a pan-cancer prognostic factor associated with recurrence and death. Elife.

[21] Frierson HF (1993). Grade and flow cytometric analysis of ploidy for infiltrating ductal carcinomas. Hum Pathol.

[22] Brestoff JR, Frater JL (2022). Contemporary challenges in clinical flow cytometry: small samples, big data, little time. J Appl Lab Med.

[23] Amrania H, Antonacci G, Chan CH, Drummond L, Otto WR, Wright NA, Phillips C (2012). Digistain: a digital staining instrument for histopathology. Opt Express.

[24] Amrania H, Drummond L, Coombes RC, Shousha S, Woodley-Barker L, Weir K, Hart W, Carter I, Phillips CC (2016). New IR imaging modalities for cancer detection and for intra-cell chemical mapping with a sub-diffraction mid-IR s-SNOM. Faraday Discuss.

[25] Amrania H, Woodley-Barker L, Goddard K (2018). Mid-infrared imaging in breast cancer tissue: an objective measure of grading breast cancer biopsies. Converg Sci Phys Oncol.

[26] Royston P, Moons KG, Altman DG, Vergouwe Y (2009). Prognosis and prognostic research: Developing a prognostic model. BMJ.

[27] Steyerberg EW (2009). Overfitting and optimism in prediction models. Clin Predict Models.

[28] McLachlan G, Do KA, Ambroise C (2005) Analyzing Microarray Gene Expression Data. ISBN:471726128.

[29] Sheltzer JM, Amon A (2011). The aneuploidy paradox: costs and benefits of an incorrect karyotype. Trends Genet.

[30] McGranahan N, Burrell RA, Endesfelder D, Novelli MR, Swanton C (2012). Cancer chromosomal instability: therapeutic and diagnostic challenges. EMBO Rep.

[31] Gazic B, Pizem J, Bracko M, Cufer T, Borstnar S, Pohar-Marinsek Z, Us-Krasovec M (2008). S-phase fraction determined on fine needle aspirates is an independent prognostic factor in breast cancer – a multivariate study of 770 patients. Cytopathology.

[32] Hatschek T, Gröntoft O, Fagerberg G, Stål O, Sullivan S, Carstensen J, Nordenskjöld B (1990). Cytometric and histopathologic features of tumors detected in a randomized mammography screening program: correlation and relative prognostic influence. Breast Cancer Res Treat.

[33] van de Vijver MJ, He YD, van’t Veer LJ, Dai H, Hart AA, Voskuil DW, Schreiber GJ, Peterse JL, Roberts C, Marton MJ, Parrish M, Atsma D, Witteveen A, Glas A, Delahaye L, van der Velde T, Bartelink H, Rodenhuis S, Rutgers ET, Friend SH, Bernards R (2002). A gene-expression signature as a predictor of survival in breast cancer. N Engl J Med.

[34] Engelhardt EG, Garvelink MM, de Haes JH, van der Hoeven JJ, Smets EM, Pieterse AH, Stiggelbout AM (2014). Predicting and communicating the risk of recurrence and death in women with early-stage breast cancer: a systematic review of risk prediction models. J Clin Oncol.

[35] Buyse M, Loi S, van’t Veer L, Viale G, Delorenzi M, Glas AM, d’Assignies MS, Bergh J, Lidereau R, Ellis P, Harris A, Bogaerts J, Therasse P, Floore A, Amakrane M, Piette F, Rutgers E, Sotiriou C, Cardoso F, Piccart MJ (2006). Validation and clinical utility of a 70-gene prognostic signature for women with node-negative breast cancer. J Natl Cancer Inst.

[36] Wittner BS, Sgroi DC, Ryan PD, Bruinsma TJ, Glas AM, Male A, Dahiya S, Habin K, Bernards R, Haber DA, Van't Veer LJ, Ramaswamy S (2008). Analysis of the MammaPrint breast cancer assay in a predominantly postmenopausal cohort. Clin Cancer Res.

[37] Sengupta AK, Gunda A, Malpani S, Serkad CPV, Basavaraj C, Bapat A, Bakre MM (2020). Comparison of breast cancer prognostic tests CanAssist Breast and Oncotype DX. Cancer Med.

[38] Thibodeau S, Voutsadakis IA (2019). Prediction of Oncotype Dx recurrence score using clinical parameters: a comparison of available tools and a simple predictor based on grade and progesterone receptor. Hematol Oncol Stem Cell Ther.

[39] National Institute for Health and Care Excellence: (2018) Tumour profiling tests to guide adjuvant chemotherapy decisions in early breast cancer. Available at: https://www.nice.org.uk/guidance/dg34/chapter/1-Recommendations. Accessed 01 August 2023

