# Correction: Performance of a novel spectroscopy-based tool for adjuvant therapy decision-making in hormone receptor-positive breast cancer: a validation study

**DOI:** 10.1007/s10549-024-07276-z

**Published:** 2024-05-05

**Authors:** R Charles Coombes, Christina Angelou, Zamzam Al-Khalili, William Hart, Darius Francescatti, Nicholas Wright, Ian Ellis, Andrew Green, Emad Rakha, Sami Shousha, Hemmel Amrania, Chris C. Phillips, Carlo Palmieri

**Affiliations:** 1 Imperial College London, South Kensington Campus, London, SW7 2AZ UK; 2 Rush Medical College, Chicago, USA; 3 Barts Cancer Institute, London, UK; 4 Nottingham University Hospital, Nottingham, UK; 5 University of Liverpool, Liverpool, UK

**Correction: Breast Cancer Research and Treatment**

10.1007/s10549-023-07229-y

In the original publication of the article, the name of the first author was published incorrectly as Charles Coombes. The corrected name should read as R Charles Coombes.

The original article has been corrected.

