# Supplementary material for: Performance of a novel spectroscopy-based tool for adjuvant therapy decision-making in hormone receptor-positive breast cancer: a validation study
Source: Breast Cancer Res Treat. 2024 Jan 20;205(2):349–58. doi: 10.1007/s10549-023-07229-y (PMC11101376; doi:10.1007/s10549-023-07229-y)
Supplement: Supplementary file 1 — Supplementary file1 (DOCX 1873 kb) [file 10549_2023_7229_MOESM1_ESM.docx]

**Supplementary Material**

## **Additional methods**

## **Tissue sample preparation**

Tissue microarrays (TMAs) representing the series have been previously described by Albanghali et al [1]. Briefly, the TMAs were constructed by removing a 0.6 mm wide core of tissue from each patient’s paraffin block. The core was taken from a pre-selected area that captured the clinical meaningful features of tumour aggressiveness. Each core was then transferred to a receiving paraffin block in a predetermined position. The Digistain procedure was performed on sections cut from the TMAs as described previously [2,3].

Reproducibility of the Digistain Index (DI) within blocks was assessed by taking six serial sections of a formalin-fixed tissue block from a single patient. The within-block standard deviation for the DI was 0·027. This translated to a 97% reproducibility of the DI within the block.

## **Statistical analysis**

DI was summarised descriptively using standard descriptive statistics (mean, median, standard deviation, minimum, and maximum value). The use of data transformations such as log or inverse was considered for non-normal distributions and the distribution of the transformed variables was summarised. The DI cut-off points were prospectively chosen for all clinical outcomes.

Kaplan-Meier curves were used to display recurrence free survival, recurrence, and overall survival for high- and low-risk patients. Median and 95% CI are reported where they can be calculated. The length of follow up is reported descriptively.

Modelling methods followed the approach recommended by Royston et al [4]. The model assumptions included: 1) Due to limited number of events, the sample was not split into training and validation, instead bootstrapping via the K-fold cross validation was performed to assess performance adjusting for over-optimism as described by Steyerberg [5]; 2) Five candidate predictors were included. Those known to be prognostic (age at diagnosis, tumour grade and size, and lymph node status) plus the novel marker of interest (DI); 3) No model selection took place; 4) Age has an approximately normal distribution and was included as a continuous predictor (odds ratios calculated based on a 10-year increase in age); 5) Tumour grade (Grade 1, 2, 3) and lymph node status (“1” for negative and “2” for 1–3 positive lymph nodes, were modelled as categorical variables with reference/dummy coding using grade/status 1 as the reference category; 6) Tumour size was modelled as a continuous predictor. Natural logs were used due to skewness.

The relationship between clinical categorical and/or continuous variables, and 5-year or 10-year clinical outcomes was analysed using Cox proportional hazards regression models. The variables considered included the DI, tumour size, grade, age at diagnosis, lymph node status, hormone receptor status and human epidermal growth factor receptor-2 status. Each variable was normalised relative to the mean and standard deviation, x_norm_ = (x – x_mean_)/σ_x,_ of the data set to make the hazard ratios scale-invariant and more readily comparable with one another. Receiver operator characteristics (ROC) curves were constructed and area under the ROC curve (AUC) calculated, with an AUC of 1 representing perfect prediction and 0.5 representing random prediction (i.e., a test of no value).

**References**

1. Albanghali M, Green A, Rakha E, et al (2016) Construction of tissue microarrays from core needle biopsies - a systematic literature review. Histopathology 68:323–332.
2. Amrania H, Antonacci G, Chan CH, et al (2010) Digistain: a digital staining instrument for histopathology. Opt Express 20:7290–7299.
3. Amrania H, Woodley-Barker L, Goddard K, et al (2018) Converg Sci Phys Oncol 4:025001.
4. Royston P, Moons KG, Altman DG, Vergouwe Y (2009) Prognosis and prognostic research: Developing a prognostic model. BMJ 338:b604.
5. Steyerberg EW (2009) Overfitting and optimism in prediction models. In: Clinical Prediction Models, pp.83-100. DOI:10.1007/978-0-387-77244-8_5.

## **Figure S1** **Digistain Prognostic Score accuracy (AUC under ROC curves) at 5 and 10 years for predicting outcomes in the total population,** **indicating corresponding PPV and NPV**. Note: risk cut-offs calculated for multivariate prognostic score. AUC=area under the curve; NPV=negative predictive value; PPV=positive predictive value; ROC=receiver operating characteristics curve.

#### Recurrence-free survival


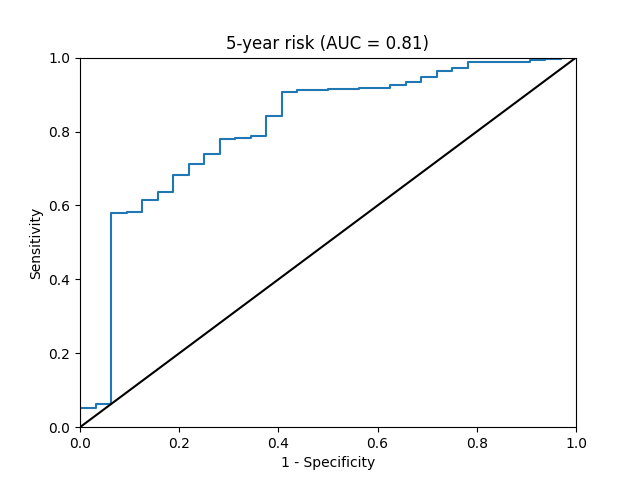

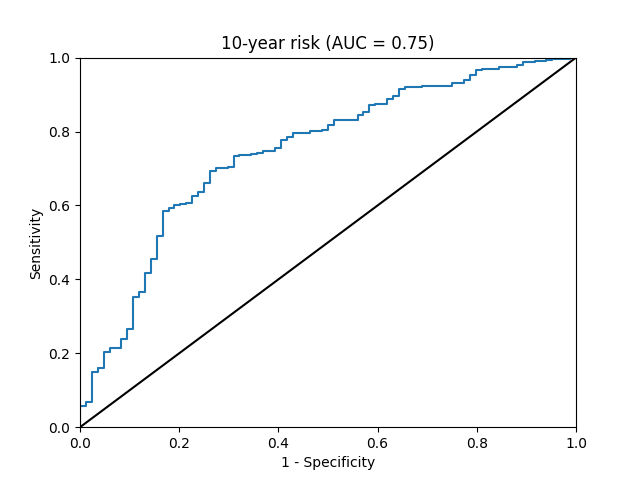


| **Year** | **High risk** | **High risk events** | **PPV** | **Low risk** | **Low risk non-events** | **NPV** | **Risk cut-off prognostic score** |
| --- | --- | --- | --- | --- | --- | --- | --- |
| 5 | 404.0 | 26.0 | 0.064 | 391.0 | 385.0 | 0.985 | 0.979 |
| 10 | 404.0 | 60.0 | 0.149 | 391.0 | 367.0 | 0.939 | 0.979 |

#### Recurrence


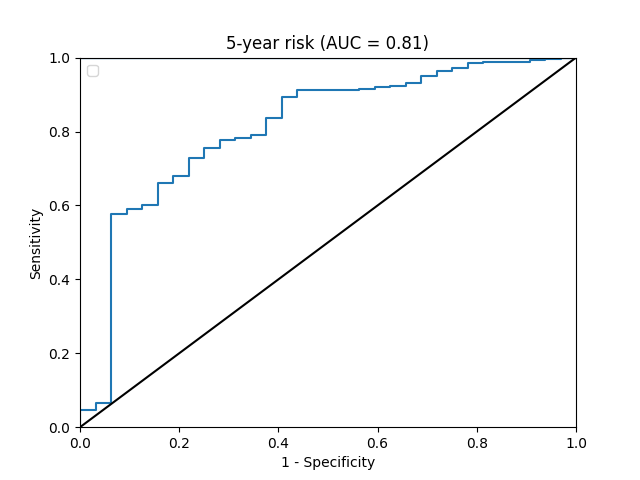

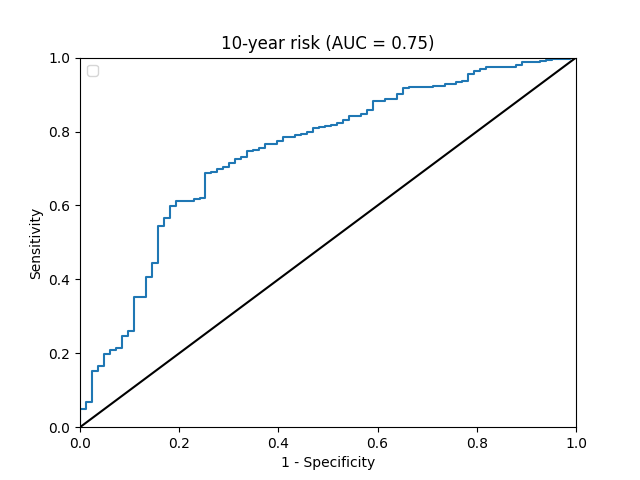


| **Year** | **High risk** | **High risk events** | **PPV** | **Low risk** | **Low risk non-events** | **NPV** | **Risk cut-off prognostic score** |
| --- | --- | --- | --- | --- | --- | --- | --- |
| 5 | 401.0 | 26.0 | 0.065 | 394.0 | 388.0 | 0.975 | 0.979 |
| 10 | 401.0 | 60.0 | 0.150 | 394.0 | 371.0 | 0.92 | 0.979 |

#### Overall survival


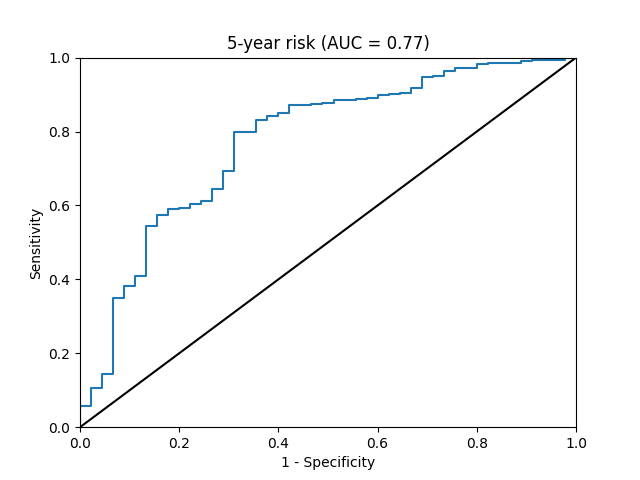

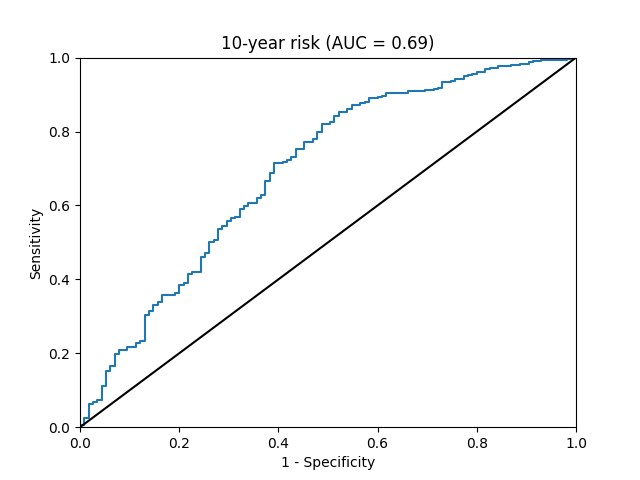


| **Year** | **High risk** | **High risk events** | **PPV** | **Low risk** | **Low risk non-events** | **NPV** | **Risk cut-off prognostic score** |
| --- | --- | --- | --- | --- | --- | --- | --- |
| 5 | 446 | 35 | 0.078 | 349 | 339 | 0.971 | 0.877 |
| 10 | 446 | 79 | 0.177 | 349 | 313 | 0.897 | 0.877 |

##

## **Figure S2** **Digistain Prognostic Score accuracy (AUC under ROC curves) at 5 and 10 years for predicting outcomes in the subgroup of lymph node-negative patients, indicating corresponding PPV and NPV.** Note: risk cut-offs calculated for multivariate prognostic score. AUC=area under the curve; NPV=negative predictive value; PPV=positive predictive value; ROC=receiver operating characteristics curve.

#### a) Recurrence-free survival


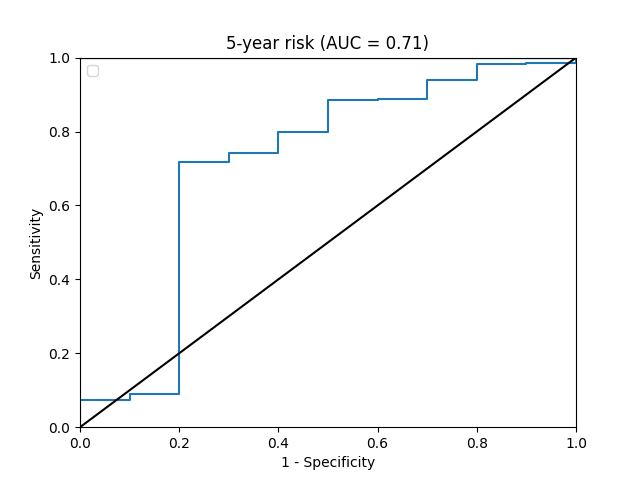

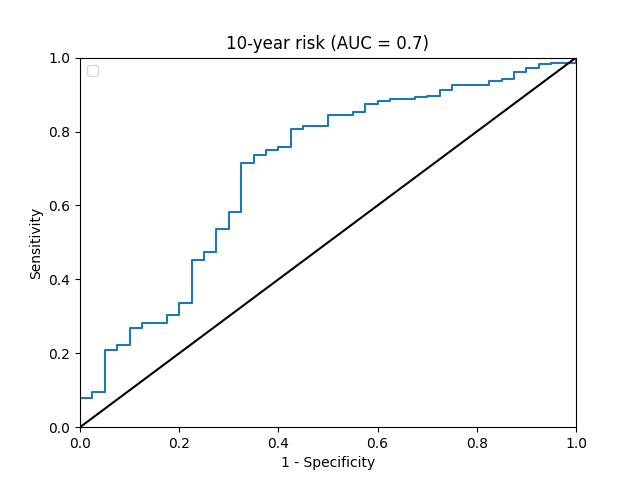


| **Year** | **High risk** | **High risk events** | **PPV** | **Low risk** | **Low risk non-events** | **NPV** | **Risk cut-off prognostic score** |
| --- | --- | --- | --- | --- | --- | --- | --- |
| 5 | 211 | 5 | 0.024 | 335 | 330 | 0.985 | 0.979 |
| 10 | 211 | 22 | 0.104 | 335 | 317 | 0.946 | 0.979 |

#### b) Recurrence


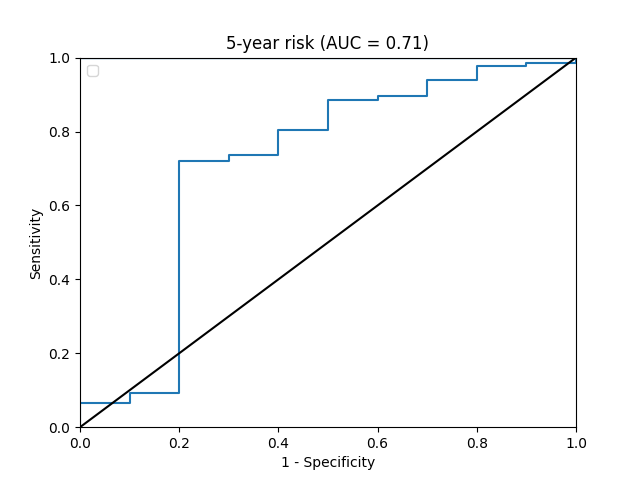

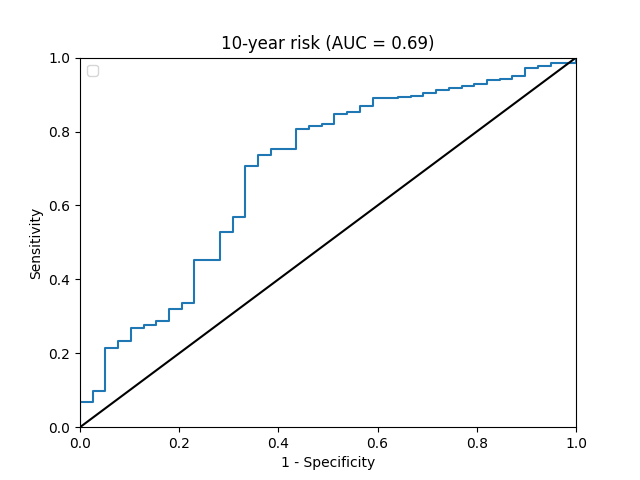


| **Year** | **High risk** | **High risk events** | **PPV** | **Low risk** | **Low risk non-events** | **NPV** | **Risk cut-off prognostic score** |
| --- | --- | --- | --- | --- | --- | --- | --- |
| 5 | 205 | 5.0 | 0.024 | 341 | 336 | 0.985 | 0.979 |
| 10 | 205 | 21.0 | 0.102 | 341 | 323 | 0.947 | 0.979 |

#### c) Overall survival


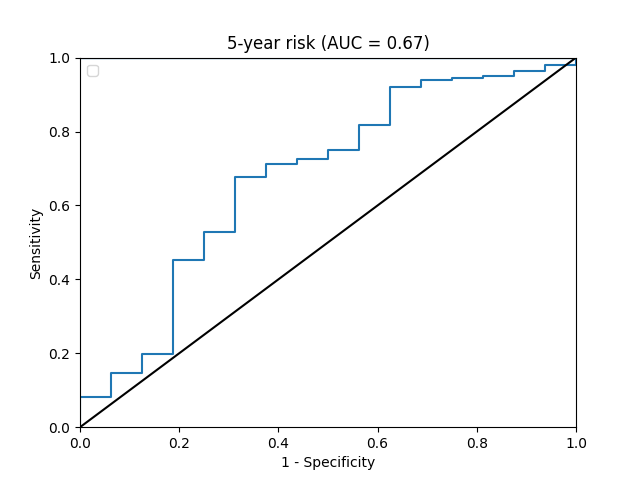

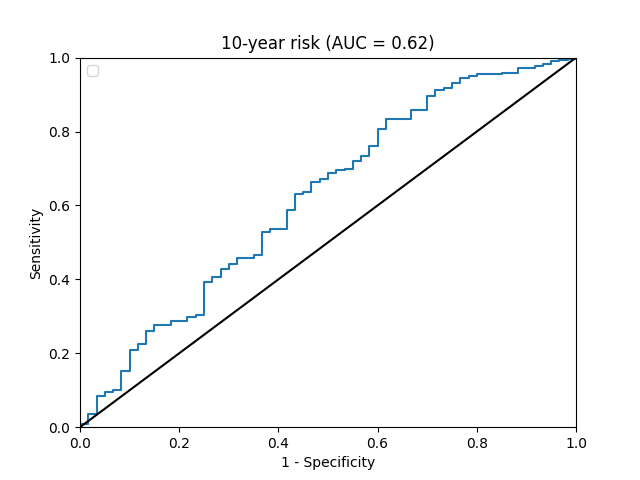


| **Year** | **High risk** | **High risk events** | **PPV** | **Low risk** | **Low risk non-events** | **NPV** | **Risk cut-off prognostic score** |
| --- | --- | --- | --- | --- | --- | --- | --- |
| 5 | 242 | 7 | 0.029 | 304 | 295 | 0.970 | 0.877 |
| 10 | 242 | 30 | 0.124 | 304 | 274 | 0.901 | 0.877 |

## **Figure S3** **Digistain Prognostic Score accuracy (AUC under ROC curves) at 5 and 10 years for predicting outcomes in the subgroup of lymph node-positive patients, indicating corresponding PPV and NPV.** Note: risk cut-offs calculated for multivariate prognostic score. AUC=area under the curve; NPV=negative predictive value; PPV=positive predictive value; ROC=receiver operating characteristics curve.

#### a) Recurrence-free survival


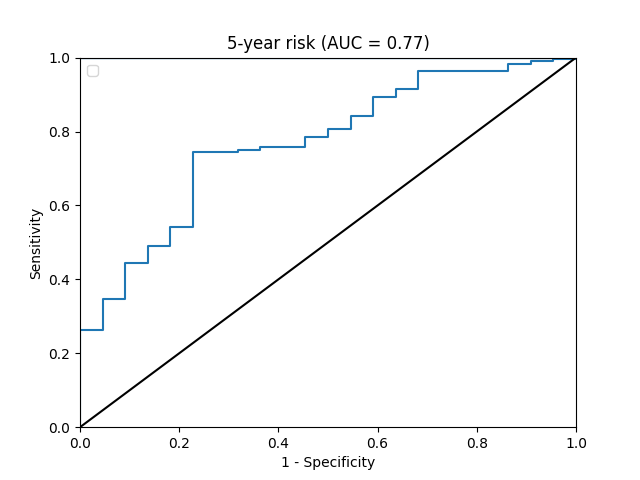

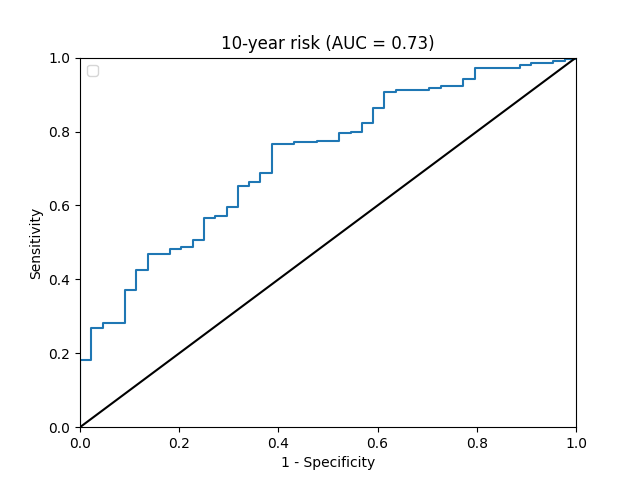


| **Year** | **High risk** | **High risk events** | **PPV** | **Low risk** | **Low risk non-events** | **NPV** | **Risk cut-off prognostic score** |
| --- | --- | --- | --- | --- | --- | --- | --- |
| 5 | 193 | 21 | 0.109 | 56 | 55 | 0.982 | 0.979 |
| 10 | 193 | 38 | 0.197 | 56 | 50 | 0.893 | 0.979 |

#### b) Recurrence


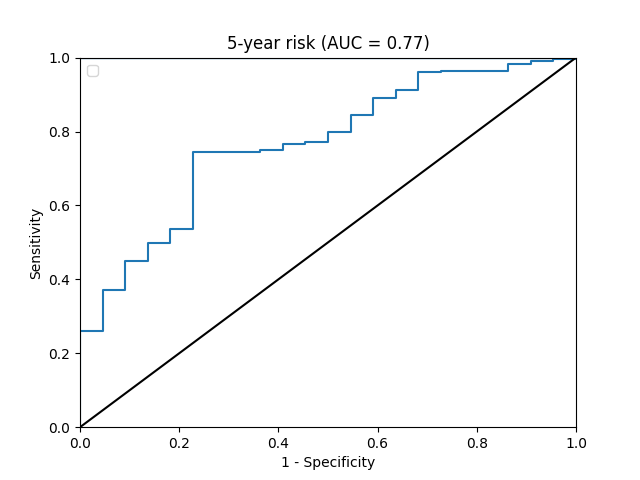

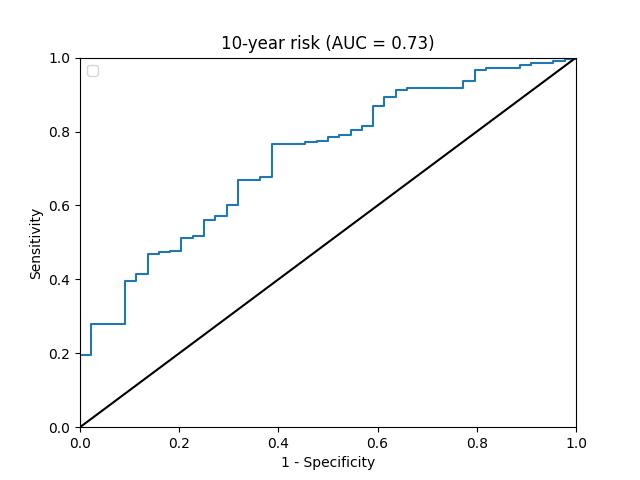


| **Year** | **High risk** | **High risk events** | **PPV** | **Low risk** | **Low risk non-events** | **NPV** | **Risk cut-off prognostic score** |
| --- | --- | --- | --- | --- | --- | --- | --- |
| 5 | 196 | 21 | 0.107 | 53 | 52 | 0.982 | 0.979 |
| 10 | 196 | 39 | 0.199 | 53 | 48 | 0.893 | 0.979 |

#### c) Overall survival


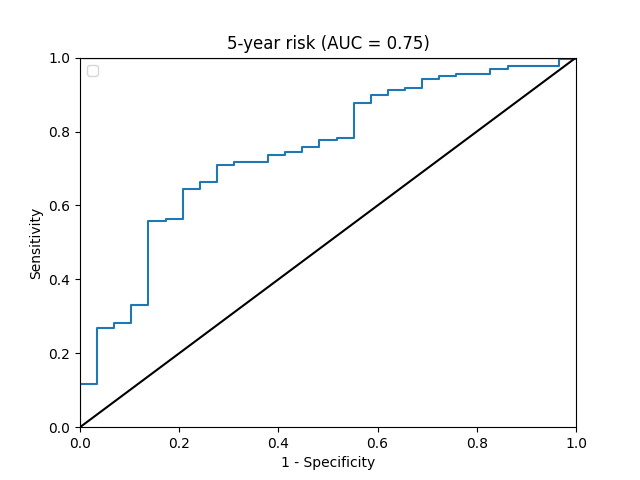

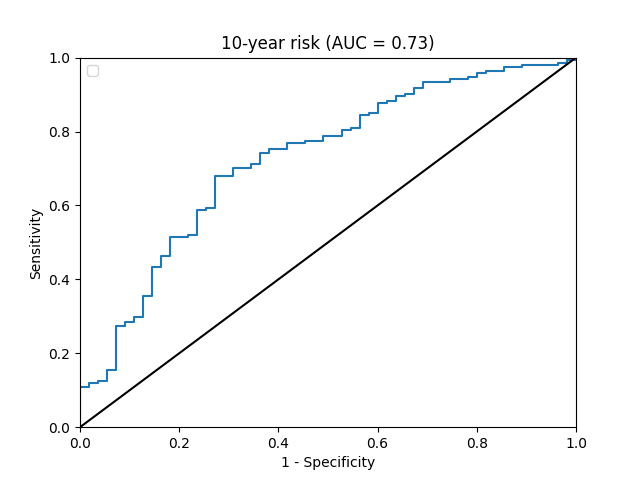


| **Year** | **High risk** | **High risk events** | **PPV** | **Low risk** | **Low risk non-events** | **NPV** | **Risk cut-off prognostic score** |
| --- | --- | --- | --- | --- | --- | --- | --- |
| 5 | 204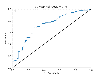 | 28 | 0.137 | 45 | 44 | 0.978 | 0.877 |
| 10 | 204 | 49 | 0.240 | 45 | 39 | 0.867 | 0.877 |

**Figure S4** **Digistain Prognostic Score accuracy (AUC under ROC curves) at 5 and 10 years for predicting outcomes in the subgroup of premenopausal patients (age ≤50 years), indicating corresponding PPV and NPV.** Note: risk cut-offs calculated for multivariate prognostic score. AUC*=*area under the curve; NPV*=*negative predictive value; PPV*=*positive predictive value; ROC*=*receiver operating characteristics curve.

#### Recurrence-free survival


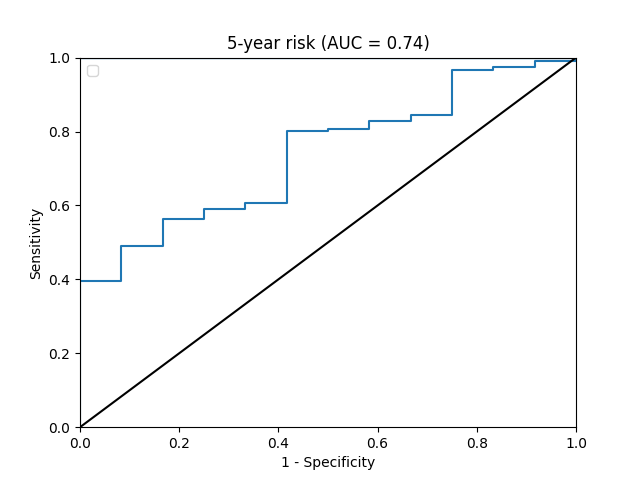

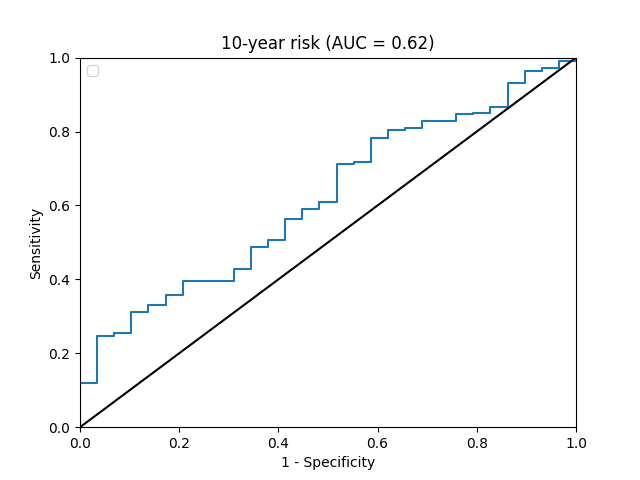


| **Year** | **High risk** | **High risk events** | **PPV** | **Low risk** | **Low risk non-events** | **NPV** | **Risk cut-off prognostic score** |
| --- | --- | --- | --- | --- | --- | --- | --- |
| 5 | 128 | 10 | 0.078 | 116 | 114 | 0.983 | 0.979 |
| 10 | 128 | 19 | 0.148 | 116 | 106 | 0.914 | 0.979 |

#### b) Recurrence


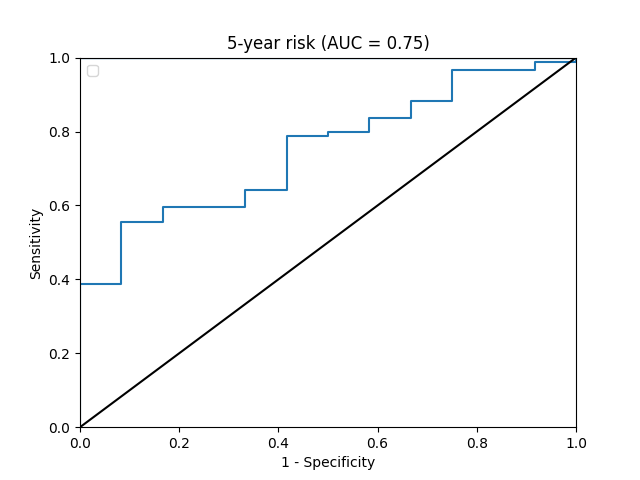

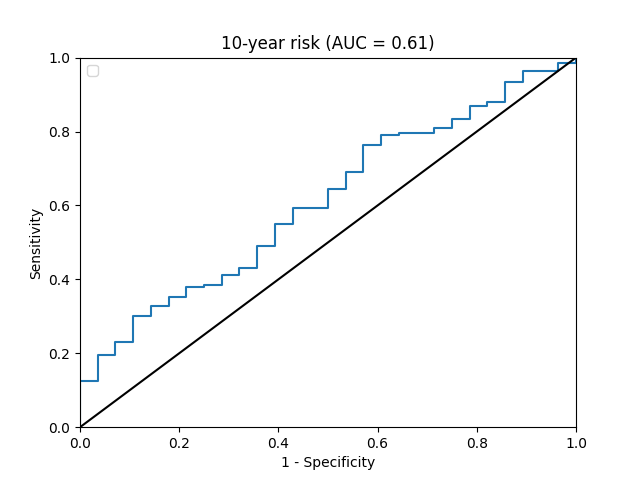


| **Year** | **High risk** | **High risk events** | **PPV** | **Low risk** | **Low risk non-events** | **NPV** | **Risk cut-off prognostic score** |
| --- | --- | --- | --- | --- | --- | --- | --- |
| 5 | 130 | 11 | 0.085 | 114 | 113 | 0.991 | 0.979 |
| 10 | 130 | 18 | 0.138 | 114 | 104 | 0.912 | 0.979 |

#### c) Overall survival


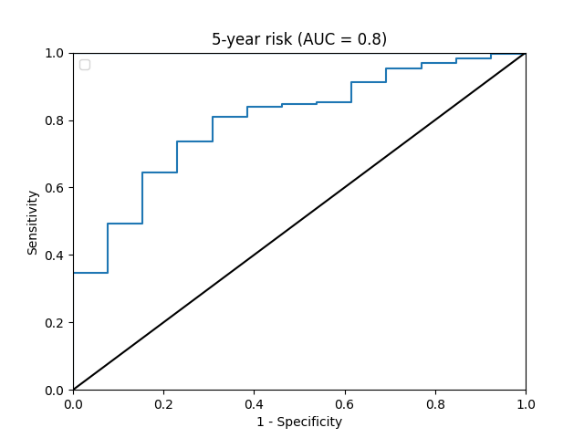

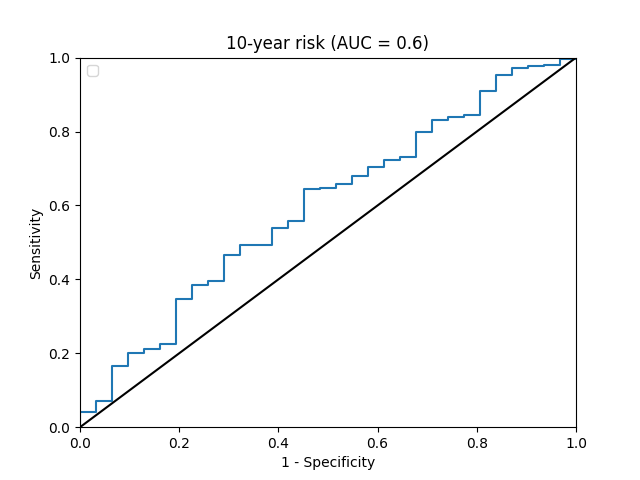


| **Year** | **High risk** | **High risk events** | **PPV** | **Low risk** | **Low risk non-events** | **NPV** | **Risk cut-off prognostic score** |
| --- | --- | --- | --- | --- | --- | --- | --- |
| 5 | 144 | 10 | 0.069 | 100 | 97 | 0.970 | 0.877 |
| 10 | 144 | 23 | 0.160 | 100 | 92 | 0.920 | 0.877 |

**Figure S5 Digistain Prognostic Score accuracy (AUC under ROC curves) at 5 and 10 years for predicting outcomes in the subgroup of postmenopausal patients (age ≥60 years), indicating corresponding PPV and NPV.** Note: risk cut-offs calculated for multivariate prognostic score. AUC*=*area under the curve; NPV*=*negative predictive value; PPV*=*positive predictive value; ROC*=*receiver operating characteristics curve.

1. Recurrence-free survival


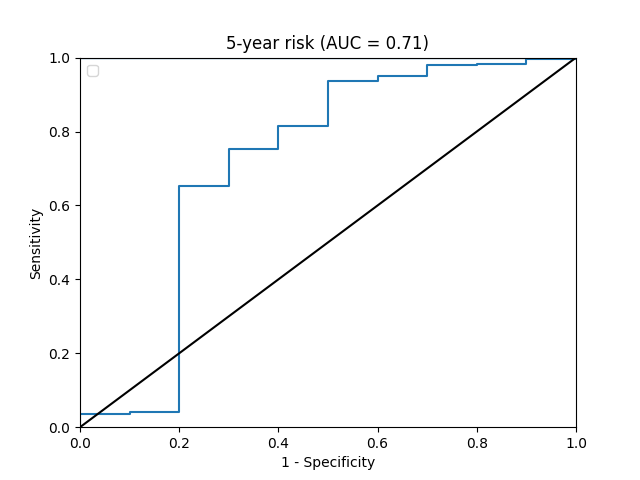

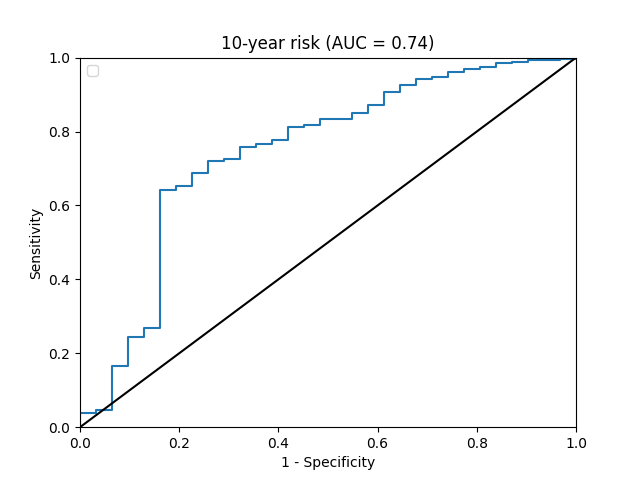


| **Year** | **High risk** | **High risk events** | **PPV** | **Low risk** | **Low risk non-events** | **NPV** | **Risk cut-off prognostic score** |
| --- | --- | --- | --- | --- | --- | --- | --- |
| 5 | 130.0 | 7.0 | 0.054 | 166.0 | 163.0 | 0.982 | 0.979 |
| 10 | 130.0 | 18.0 | 0.138 | 166.0 | 153.0 | 0.922 | 0.979 |

#### Recurrence


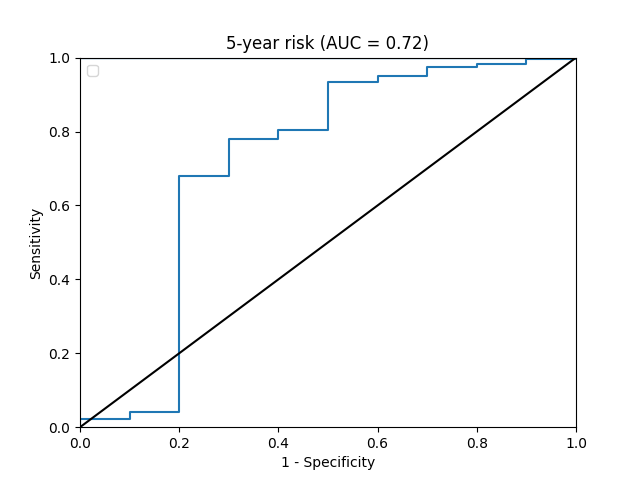

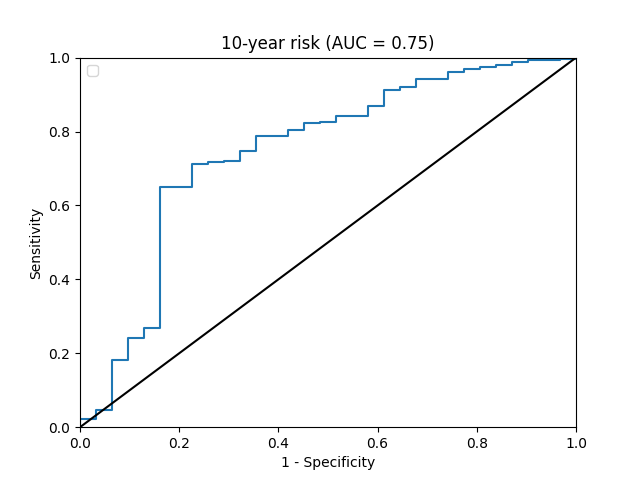


| **Year** | **High risk** | **High risk events** | **PPV** | **Low risk** | **Low risk non-events** | **NPV** | **Risk cut-off prognostic score** |
| --- | --- | --- | --- | --- | --- | --- | --- |
| 5 | 126.0 | 7.0 | 0.056 | 170.0 | 167.0 | 0.982 | 0.979 |
| 10 | 126.0 | 18.0 | 0.143 | 170.0 | 157.0 | 0.924 | 0.979 |

#### Overall survival


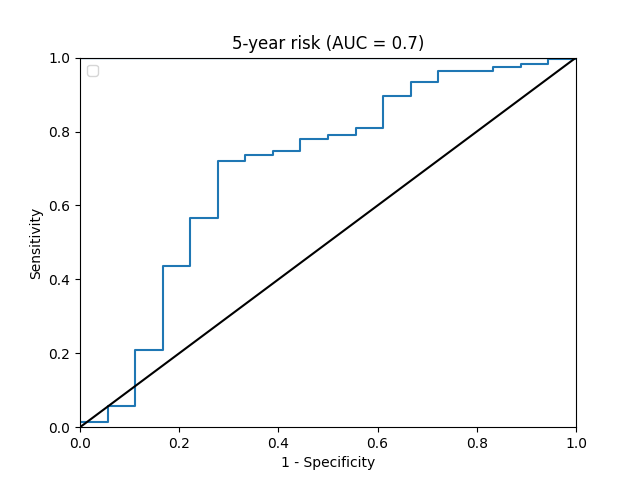

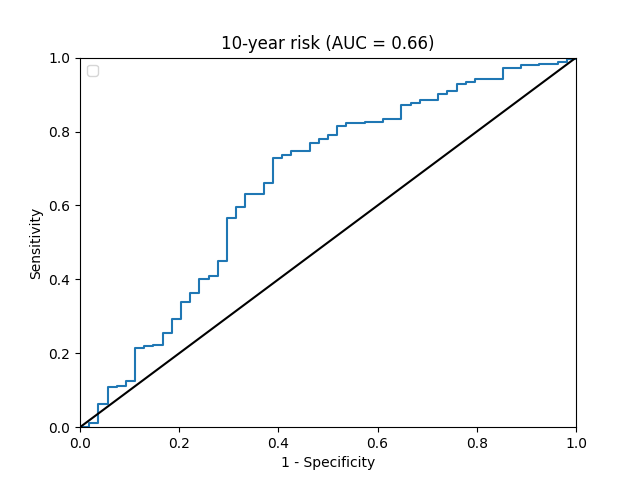


| **Year** | **High risk** | **High risk events** | **PPV** | **Low risk** | **Low risk non-events** | **NPV** | **Risk cut-off prognostic score** |
| --- | --- | --- | --- | --- | --- | --- | --- |
| 5 | 149.0 | 12.0 | 0.081 | 147.0 | 141.0 | 0.959 | 0.877 |
| 10 | 149.0 | 31.0 | 0.208 | 147.0 | 124.0 | 0.844 | 0.877 |

**Figure S6** **Kaplan-Meier curves indicating the event distribution over time for recurrence-free survival (A), recurrence (B), and overall survival (C) based on Digistain Prognostic Score classification for high and low risk in subgroups of 1) lymph node-negative, 2) lymph node-positive 3) premenopausal, and 4) postmenopausal patients.**

1. Lymph node-negative patients

| 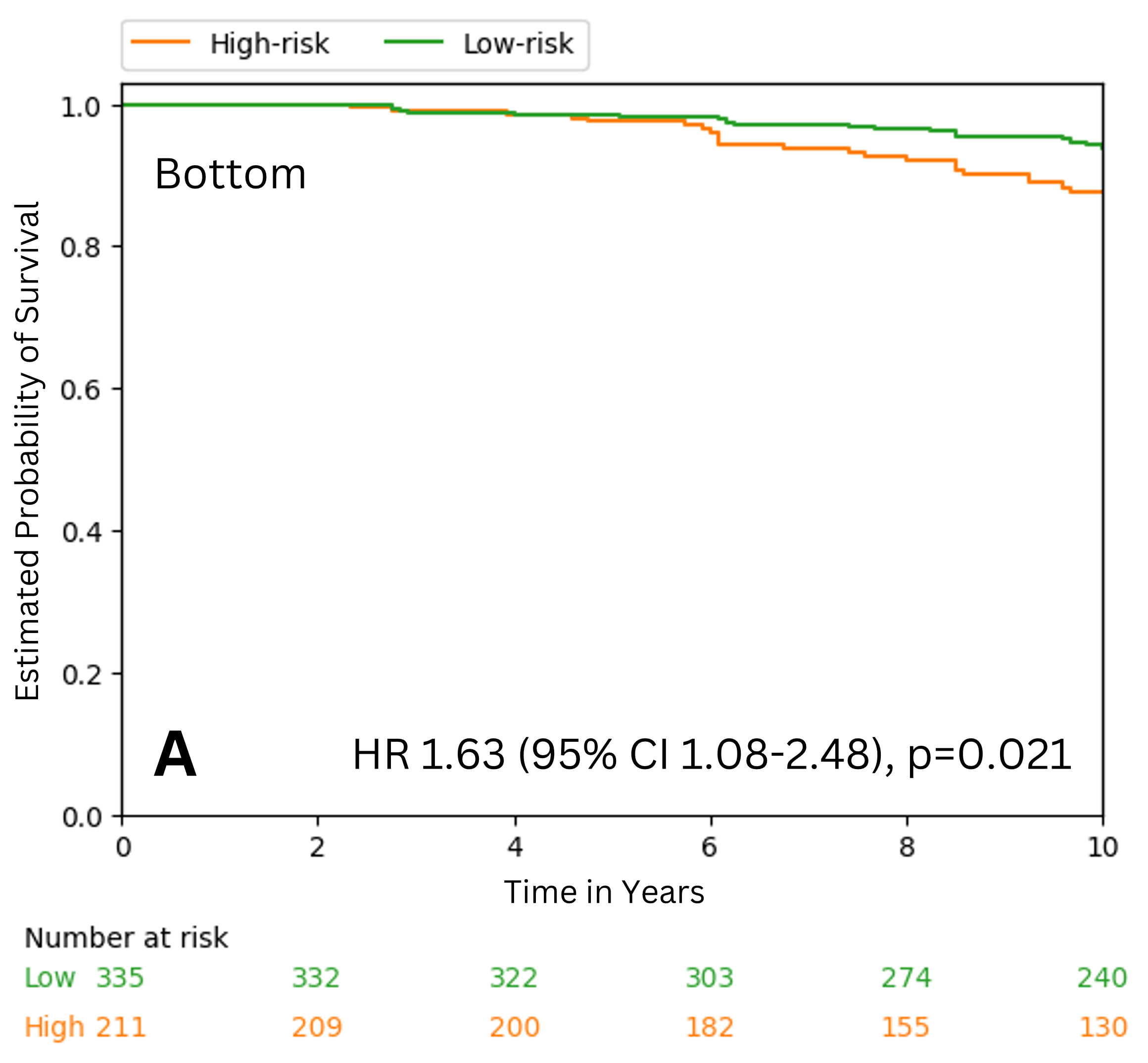 | 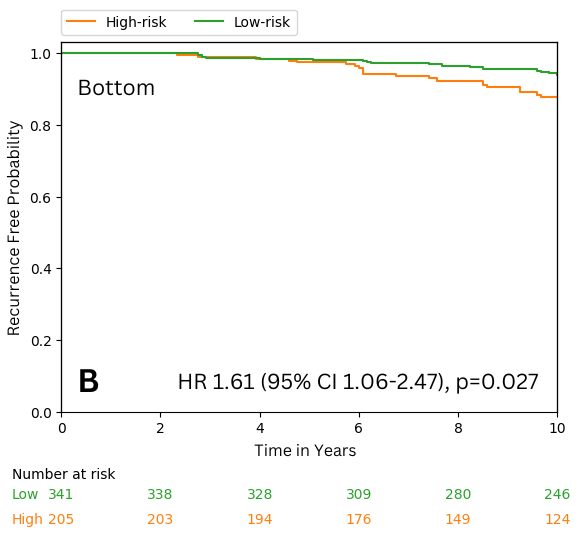 | 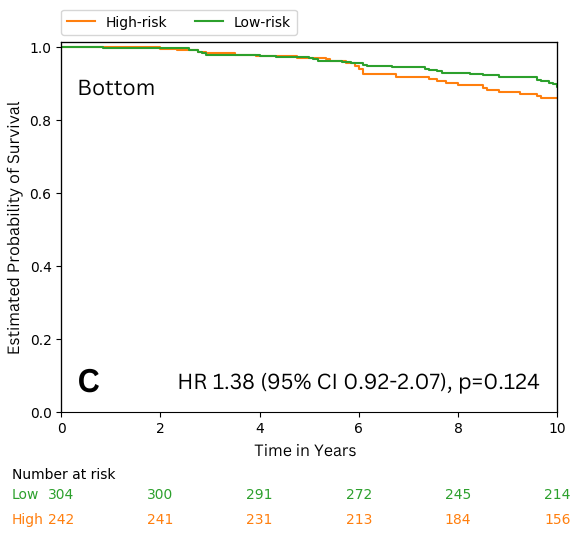 |
| --- | --- | --- |

1. Lymph node-positive patients

| 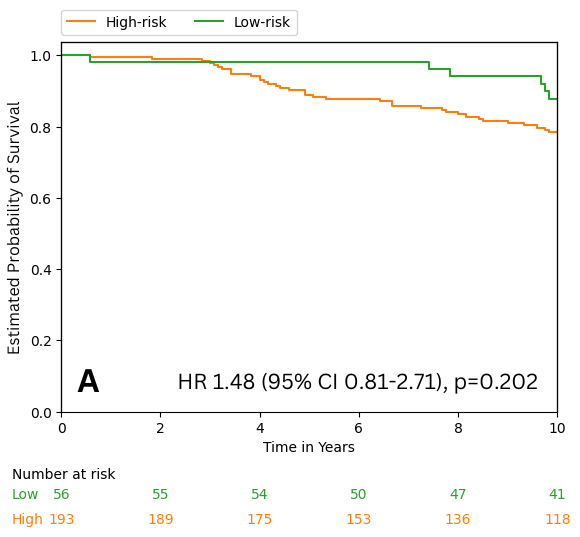 | 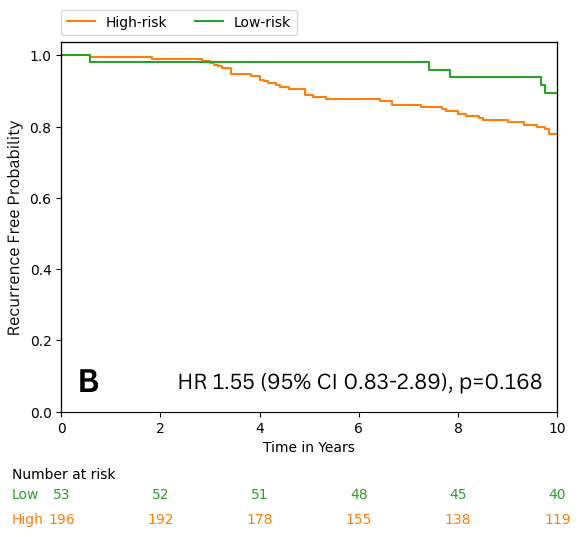 | 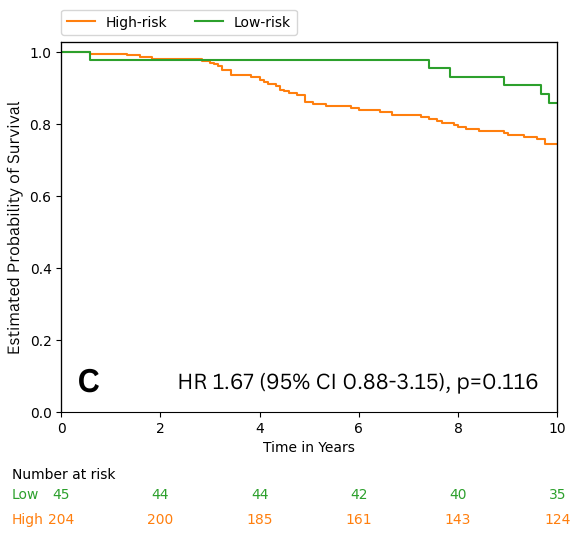 |
| --- | --- | --- |

1. Premenopausal patients (age ≤50 years)

| 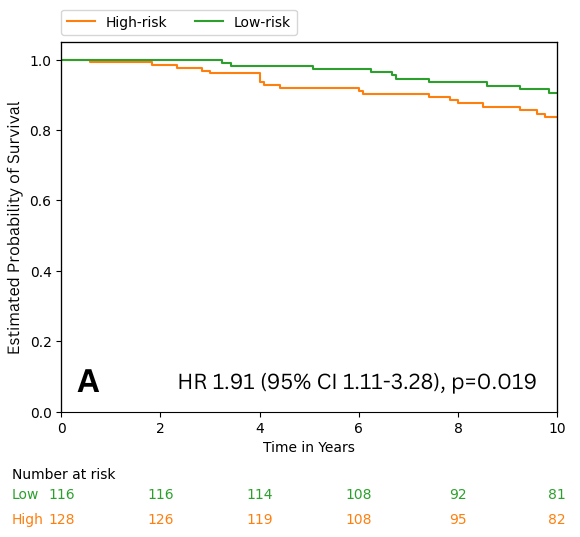 | 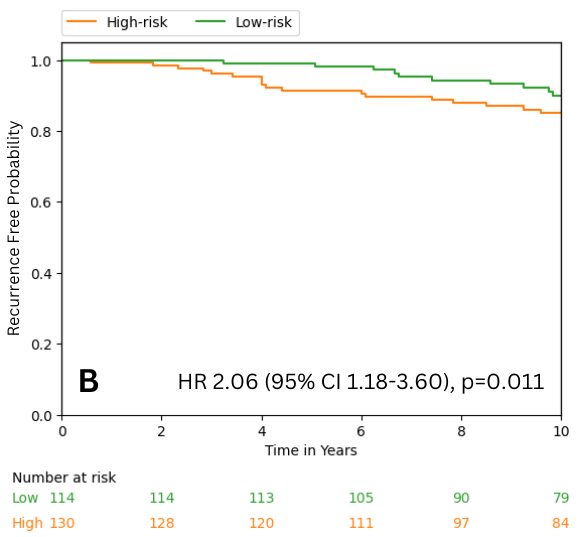 | 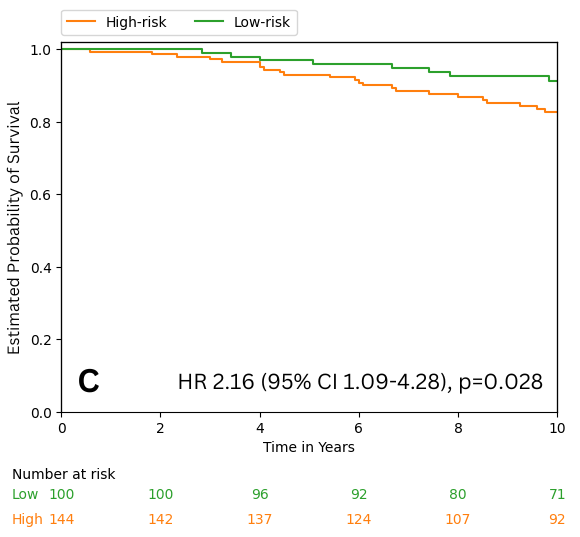 |
| --- | --- | --- |

1. Postmenopausal patients (age ≥60 years)

| 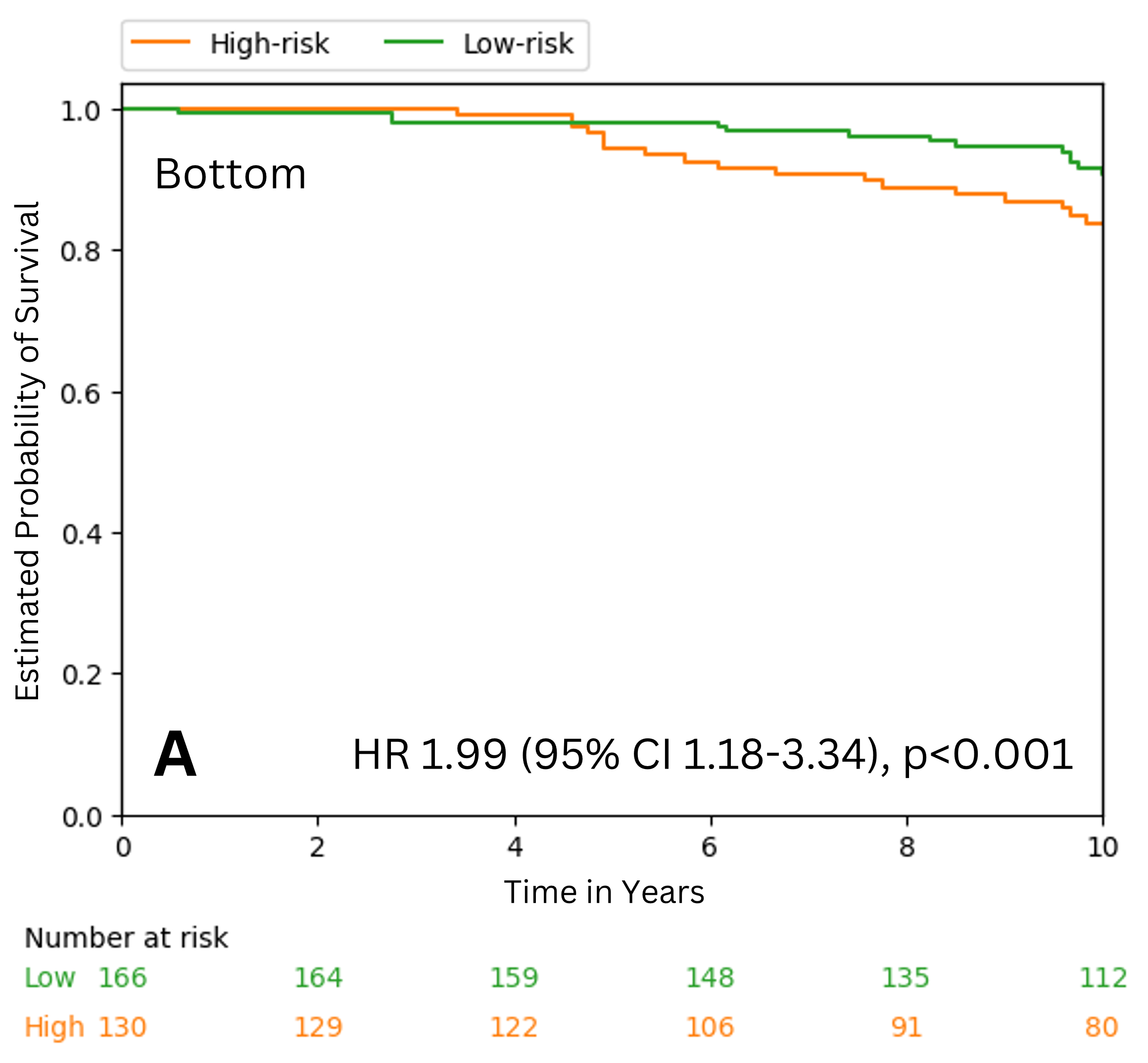 | 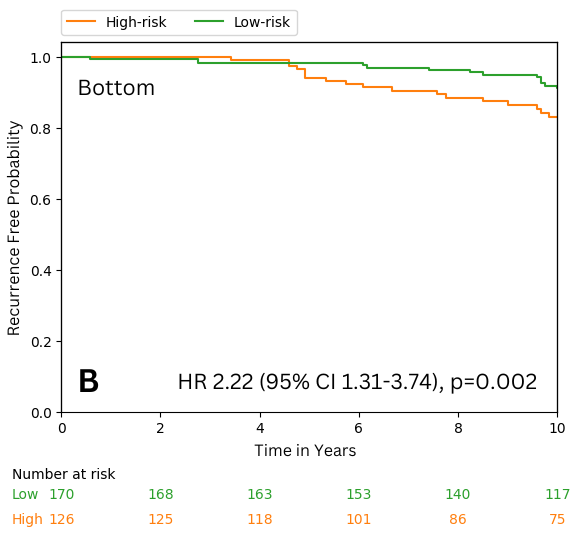 | 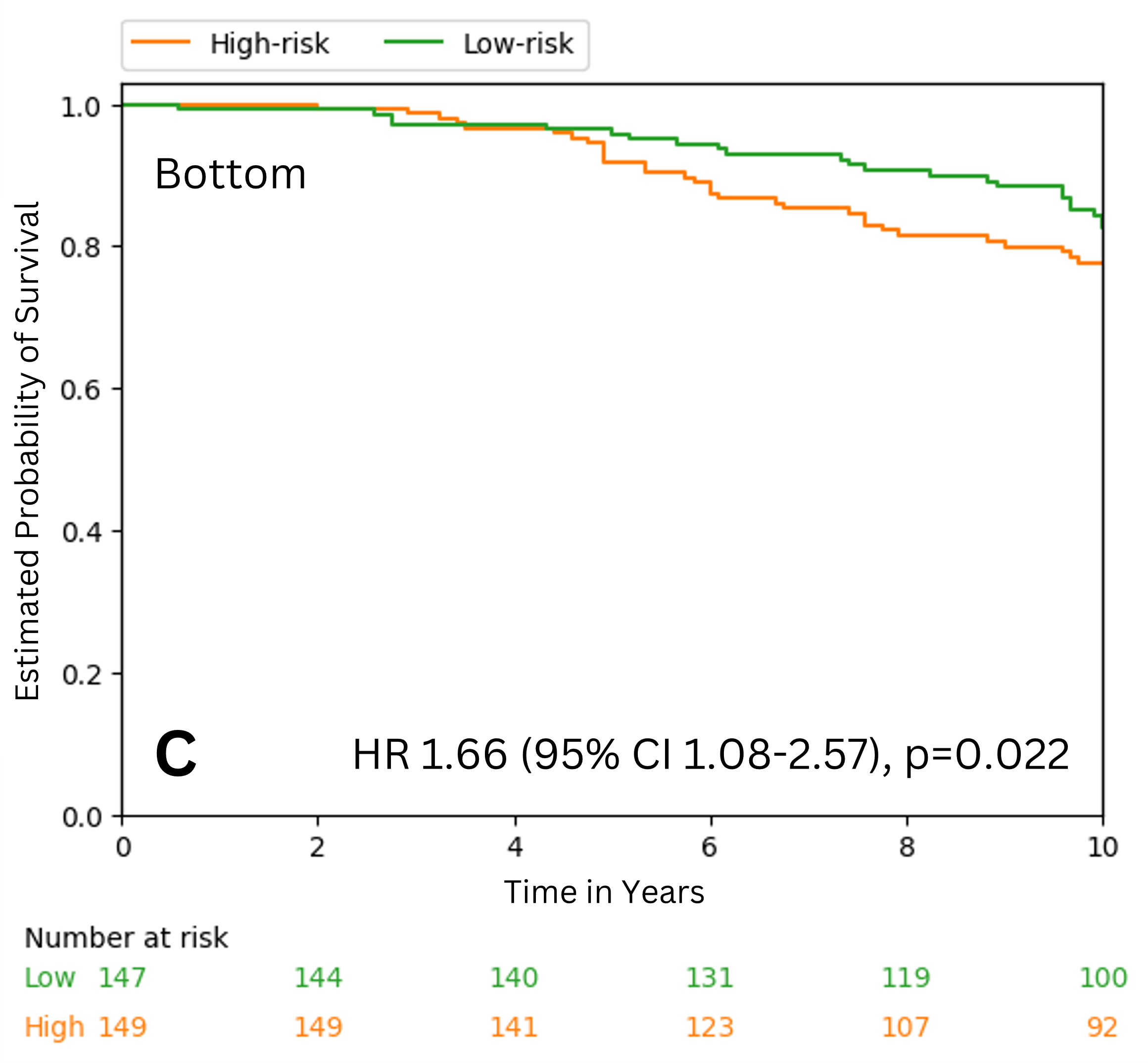 |
| --- | --- | --- |

**Table S1 Hazard ratio for recurrence-free survival, recurrence, and overall survival in the pre- and postmenopausal subgroups by lymph-node status according to Digistain Prognostic Score-based risk multivariable model high-low classification.**

|  | **Premenopausal** | | **Postmenopausal** | |
| --- | --- | --- | --- | --- |
|  | **Lymph node negative** | **Lymph node positive** | **Lymph node negative** | **Lymph node positive** |
|  | **Hazard ratio (95% CI)** | | | |
| **Recurrence-free survival** | 1.82 (0.89–3.70) *P* = 0.099 | 2.05 (0.76–5.49) *P* = 0.154 | 1.48 (0.76–2.90) *P* = 0.250 | 1.17 (0.46–2.99) *P* = 0.743 |
| N - low risk | 84 (56.76%) | 32 (33.33%) | 114 (50.89%) | 18 (25.00%) |
| N - high risk | 64 (43.24%) | 64 (66.67%) | 110 (49.11%) | 54 (75.00%) |
| **Recurrence** | 1.85 (0.90–3.80) *P* = 0.096 | 2.53 (0.86–7.41) *P* = 0.091 | 1.69 (0.85–3.35) *P* = 0.134 | 1.15 (0.45–2.93) *P* = 0.775 |
| N - low risk | 85 (57.44%) | 29 (30.21%) | 116 (51.79%) | 17 (23.61%) |
| N - high risk | 63 (42.56%) | 67 (69.79%) | 108 (48.21%) | 55 (76.39%) |
| **Overall survival** | 2.39 (0.97–5.87) *P* = 0.057 | 1.42 (0.48–4.27) *P* = 0.052 | 1.16 (0.67–2.00) *P* = 0.593 | 1.12 (0.80–6.71) *P* = 0.126 |
| N - low risk | 75 (50.68%) | 25 (26.04%) | 93 (41.52%) | 14 (19.44%) |
| N - high risk | 73 (49.32%) | 71 (73.96%) | 131 (58.48%) | 58 (80.56%) |
